# Subtle genetic changes enhance virulence of methicillin resistant and sensitive *Staphylococcus aureus*

**DOI:** 10.1186/1471-2180-7-99

**Published:** 2007-11-06

**Authors:** Sarah K Highlander, Kristina G Hultén, Xiang Qin, Huaiyang Jiang, Shailaja Yerrapragada, Edward O Mason, Yue Shang, Tiffany M Williams, Régine M Fortunov, Yamei Liu, Okezie Igboeli, Joseph Petrosino, Madhan Tirumalai, Akif Uzman, George E Fox, Ana Maria Cardenas, Donna M Muzny, Lisa Hemphill, Yan Ding, Shannon Dugan, Peter R Blyth, Christian J Buhay, Huyen H Dinh, Alicia C Hawes, Michael Holder, Christie L Kovar, Sandra L Lee, Wen Liu, Lynne V Nazareth, Qiaoyan Wang, Jianling Zhou, Sheldon L Kaplan, George M Weinstock

**Affiliations:** 1 Department of Molecular Virology and Microbiology, Baylor College of Medicine, Houston, TX, USA; 2 Department of Pediatrics, Baylor College of Medicine, Texas Children's Hospital, Houston, TX, USA; 3 Human Genome Sequencing Center, Baylor College of Medicine, Houston, TX, USA; 4 Department of Molecular and Human Genetics, Baylor College of Medicine, Houston, TX, USA; 5 University of Houston-Downtown, Houston, TX, USA; 6 University of Houston, Houston, TX, USA

## Abstract

**Background:**

Community acquired (CA) methicillin-resistant *Staphylococcus aureus *(MRSA) increasingly causes disease worldwide. USA300 has emerged as the predominant clone causing superficial and invasive infections in children and adults in the USA. Epidemiological studies suggest that USA300 is more virulent than other CA-MRSA. The genetic determinants that render virulence and dominance to USA300 remain unclear.

**Results:**

We sequenced the genomes of two pediatric USA300 isolates: one CA-MRSA and one CA-methicillin susceptible (MSSA), isolated at Texas Children's Hospital in Houston. DNA sequencing was performed by Sanger dideoxy whole genome shotgun (WGS) and 454 Life Sciences pyrosequencing strategies. The sequence of the USA300 MRSA strain was rigorously annotated. In USA300-MRSA 2658 chromosomal open reading frames were predicted and 3.1 and 27 kilobase (kb) plasmids were identified. USA300-MSSA contained a 20 kb plasmid with some homology to the 27 kb plasmid found in USA300-MRSA. Two regions found in US300-MRSA were absent in USA300-MSSA. One of these carried the arginine deiminase operon that appears to have been acquired from *S. epidermidis*. The USA300 sequence was aligned with other sequenced *S. aureus *genomes and regions unique to USA300 MRSA were identified.

**Conclusion:**

USA300-MRSA is highly similar to other MRSA strains based on whole genome alignments and gene content, indicating that the differences in pathogenesis are due to subtle changes rather than to large-scale acquisition of virulence factor genes. The USA300 Houston isolate differs from another sequenced USA300 strain isolate, derived from a patient in San Francisco, in plasmid content and a number of sequence polymorphisms. Such differences will provide new insights into the evolution of pathogens.

## Background

Community acquired methicillin-resistant *Staphylococcus aureus *(CA-MRSA) infections are increasing worldwide [1,2]. Although the emergence is concurrent, genetically distinct clones have been reported from different continents [3-6]. In the United States, two clones, USA300 and USA400 have been associated with the majority of CA-MRSA infections [7]. However, USA300 is currently being reported as the predominant cause of both adult and pediatric CA-MRSA infections in many states [8-12]. At Texas Children's Hospital (TCH) in Houston, Texas, we have observed a continuous increase of CA-*S. aureus *infections, predominantly CA-MRSA, in a prospective surveillance study initiated in 2001 [13]. Skin and soft tissue infections (SSTI) represented 94%; 58% of SSTI patients were hospitalized. We have also reported an increased number and severity of invasive cases, *e.g*. complicated pneumonia, osteomyelitis, pyomyositis, myositis and severe sepsis syndrome associated with the predominance of clone USA300 [9,14-18]. Similar observations have been described at other institutions across the United States [19]. Recent reports suggest that USA300 is more virulent than other MRSA strains [15,20,21]. USA300 is genetically related to the nosocomial MRSA strain COL, which emerged in the 1960s in Europe following the introduction of methicillin. Both of these strains are likely descendents of a methicillin-susceptible ancestral strain [22,23].

**Figure 1:**
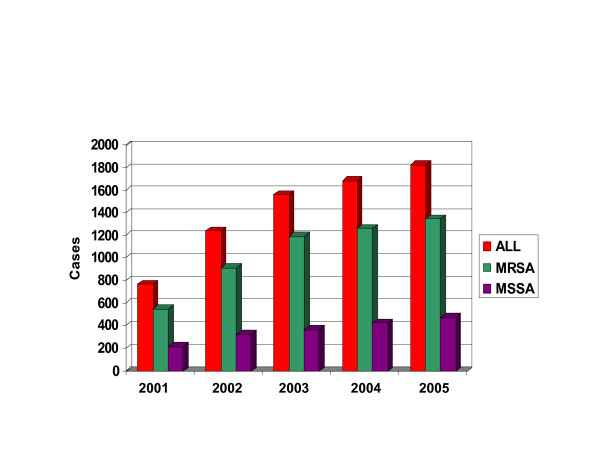
Histogram of the prevalence of community-acquired MR and MS strains of *S. aureus *at TCH over a four-year study. All cases are shown in red, MR cases in green and MS cases in purple.

**Figure 2:**
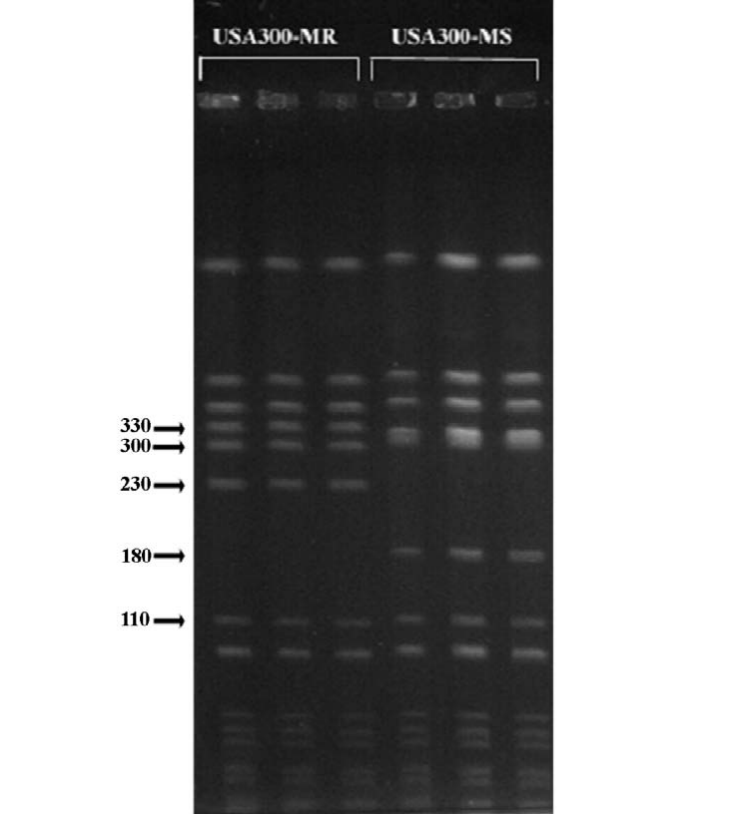
PFGE of *Sma *I-digested USA300-HOU-MR and USA300-HOU-MS. Replicates of MR (left) and MS (right) profiles are shown. Molecular weight markers are shown in kilobases.

Approximately 75% of the CA-*S. aureus *strains at TCH are MRSA; 95% of these are USA300. In contrast to the homogeneity of CA-MRSA, CA methicillin-susceptible *S. aureus *(MSSA) from the same population are more heterogeneous; USA300 represented approximately 25% of these strains between 2000–2002 [9]. We continue to note a greater absolute increase in the number of CA-MRSA isolates, being virtually all USA300-MR, compared with CA-MSSA among children evaluated at TCH (Figures 1 and 2).

At the time of this writing, the complete genomes of eleven MRSA and MSSA clinical strains, of both community and hospital origin, plus a bovine *S. aureus *strain have been deposited in the GenBank database. Recently, the genome sequence of a multi-resistant USA300-MR isolate (FPR3757) from an abscess of an HIV-positive intravenous drug user was reported [24]. We present the sequence of a USA300-MR isolate from an otherwise healthy pediatric patient (USA300-HOU) and compare it to the genomes of FPR3757 and a USA300-MS isolate from TCH. We postulate that comparison of the genomes of USA300-MR and USA300-MS will provide clues to the enhanced ability of USA300-HOU-MR to spread and cause infections among children compared to other *S. aureus *clones circulating in Houston.

## Results

### Features of the USA300-HOU-MR genome

**Figure 3:**
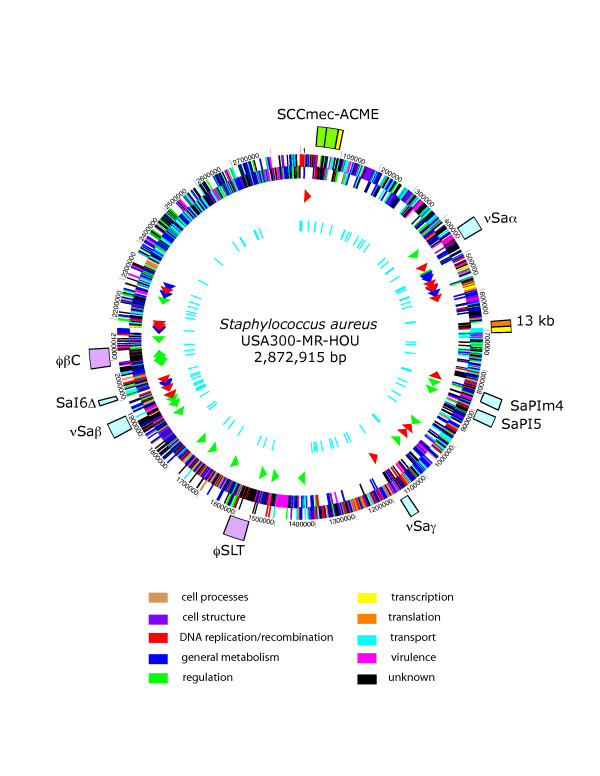
Chromosomal map of USA300-HOU-MR with landmarks indicating the SCC*mec*-ACME region (outer circle, green), pathogenicity islands (outer circle, light blue), prophage (outer circle, lavender), the 13 kb insertion sequence (outer circle, orange), and two regions of sequence not found in USA300-HOU-MS (outer circle, yellow). ORFs on both strands are represented by the second circle and are colored according to functional categories as follows and as shown in the key: cell processes, tan; cell structure, violet; DNA replication and recombination, red; general metabolism, blue; regulation, green; transcription, yellow; translation, orange; transport, cyan; virulence, fuchsia; unknown, black. The third circle shows RNAs: rRNAs, blue; tRNAs, red; ncRNAs, green. The inner circle shows the location of SNPs between USA300-HOU-MR and FPR3757.

**Figure 4:**
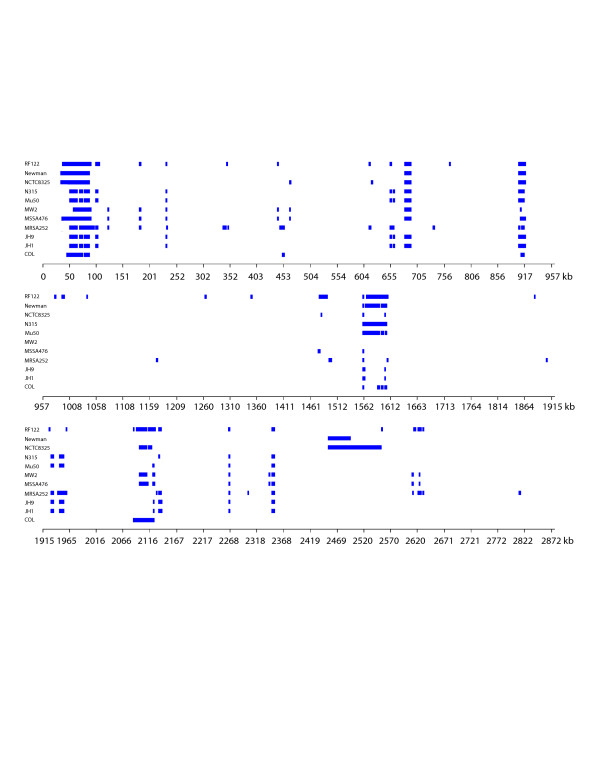
Linear representation of BLASTN comparison of *S. aureus *strains to USA300-HOU-MR. Strains are listed on the y-axis and the x-axis shows the USA300-HOU-MR coordinates. Bars represent regions of at least 50 bp in length present in USA300-HOU-MR but absent in the other strains.

The USA300-HOU-MR genome is 2,872,915 bp in length and was annotated to encode 2658 proteins, 59 tRNAs (plus one pseudogene), 5 ribosomal RNA operons and 21 non-coding RNAs (Table 1). A circular representation of the sequenced genome is shown in Figure 3. To identify DNA regions unique to USA300-HOU-MR, two-way BLASTN was performed against each of the other complete staphylococcal genomes (Figure 4). USA300-HOU-MR carries the staphylococcal cassette chromosome *mec *(SCC*mec*) type IVa methicillin resistance cassette. In addition to SCC*mec *IVa, the strain also contains a ca. 31 kb region of DNA from 57860 to 88845 that is unique only to USA300-HOU-MR, USA300-FPR3757 and to *S. epidermidis *ATCC12228 (Additional file 1) [25]. This region maps immediately downstream of SCC*mec *and the entire element (34173 to 88845) is flanked by an IS*431mec *transposase gene and a possible transposase gene. The region downstream of SCC*mec *carries genes encoding an arginine deiminase operon, a Crp family transcriptional regulator, an APC family arginine/ornithine antiporter, and an arginine repressor. In USA300-FPR3757, this region is called ACME, for arginine catabolic mobile element [24]. Downstream of the arginine element we also annotated genes encoding a universal stress protein, a lysophospholipase, a possible S-adenosyl-L-methionine (SAM)-dependent methyltransferase, an ABC superfamily ATP binding cassette transporter, a P-ATPase copper transporter, a possible lipoprotein and an integrase. The ABC transporter proteins had top BLAST hits to nickel, dipeptide or oligopeptide transporters in other species and it was annotated as an oligopeptide transporter by Diep, et al. [24]. ClustalW analyses of the individual components of these transporters indicated a clustering of nickel-specific sequences with those from USA300-HOU-MR (data not shown). Thus, we believe that this transporter is likely involved in nickel acquisition. InterProScan, PFP and PSORTB, SecretomeP2.0 were used to augment the annotation data obtained by BLASTP and CDD. Based on this analysis, the SCC*mec*-ACME region encodes four non-classically secreted proteins and three other extracellular proteins. The region also encodes a possible endonuclease, possible tRNA ligases, RNA polymerases and a primase. We also identified a number of ORFs that were not annotated in USA300-FPR3757; these were hypothetical or conserved hypothetical proteins.

**Table 1:** Riboswitches and small non-coding RNAs in USA300-HOU-MR

**Start**	**Stop**	**Definition**
**Riboswitches**		
1,556,144	1,556,024	FMN riboswitch
1,899,283	1,899,148	FMN riboswitch
1,653,467	1,653,370	GcvT riboswitch
2,272,812	2,272,596	GlmS riboswitch
1,430,494	1,430,669	lysine riboswitch
1,783,037	1,782,862	lysine riboswitch
436,036	436,135	purine riboswitch
893,057	893,160	SAM riboswitch
1,025,974	1,026,102	YybP-YkoY riboswitch
		
**Small ncRNAs**		
1,736,694	1,736,363	6S RNA
508,051	508,326	bacterial signal recognition particle RNA
1,930,023	1,929,680	noncoding RNA SprA
1,946,423	1,945,949	noncoding RNA SprB
1,959,339	1,958,106	noncoding RNA SprC
1,958,812	1,958,861	noncoding RNA SprC
2,087,533	2,086,851	noncoding RNA SprD
2,088,540	2,089,062	noncoding RNA SprE
2,090,767	2,090,977	noncoding RNA SprF/SprG
1,516,390	1,515,994	ribonuclease P (RNase P)
2,147,378	2,147,086	RNAIII
868,423	868,737	tmRNA SsrA

### Pathogenicity islands and prophages

More than fifteen different pathogenicity islands have been identified in the sequenced *S. aureus *genomes [25,26]. SaPIs are mobile genetic elements that carry about half of the known *S. aureus *toxin or virulence factor genes, and while strains contain different combinations of the islands, no strain appears to carry more than one copy of each type [27]. USA300-HOU-MR contains six, SaPI5, SaPIm4, SaPI6Δ, *v*Saα, *v*Saβ, and *v*Saγ. These encode enterotoxins, extotoxins and serine proteases as well as leukotoxin, hemolysin, exfoliatin, and epidermin (Figure 3 and Table 2). MW2, another community-acquired MRSA isolate contains the same complement of islands, while, COL, Mu50 and RF122 contain six islands each, though in different combinations.

**Table 2:** Pathogenicity islands in completed genomes of *S. aureus*

**ISLAND**	**VIRULENCE GENE PRODUCTS**	**USA300 MRSA**	**Newman**	**NCTC 8325**	**COL**	**MW2**	**MSSA 476**	**N315**	**Mu50**	**JH1**	**JH9**	**RF122**	**MRSA 252**
SaPI1	TSST, SEK, SEL, Ear	-	-	-	-	-	-	-	-	-	-	-	-
SaPI2	TSST, exfoliatin	-	-	-	-	-	-	-	-	-	-	-	-
SaPI3	SEB, SEK, SEQ, Ear	-	-	-	+	-	-	-	-	-	-	-	-
SaPI4	None	-	-	-	-	-	-	-	+	-	-	-	+
SaPI5	SEK, SEQ, Ear	+	-	-	-	+	-	-	-	-	-	-	-
SaPIbov1	TSST, SEK, SEL	-	-	-	-	-	-	-	-	-	-	+	-
SaPIbov2	Bap adhesin	-	-	-	-	-	-	-	-	-	-	-	-
SaPIm4	ferrichrome transporter	+	+	+	+	-	-	+	+	+	+	+	-
SaPImw2	Ear, SEC4, SEL2	-	-	-	-	+	-	-	-	-	-	-	-
SaPIn1/m1	TSST, SEL, SEC3	-	-	-	-	-	-	+	+	-	-	-	-
SaPI122	multidrug exporter	-	-	-	-	-	-	-	-	-	-	+	-
SaPI6Δ	None	+	+	+	+	+	+	-	-	-	-	-	-
νSaα	SET exotoxins, lipoproteins	+	+	+	+	+	+	+	+	+	+	+	+
νSaβ	serine proteases, leukotoxin D & E, enterotoxins, epidermin	+^a^	+^a^	+^a^	+^a^	+^a^	+^a, b^	+^c^	+^c^	+^c^	+^c^	+	+^b^
νSaγ	SET exotoxins, exfoliatin, alpha-hemolysin	+	+	+	+	+	+	+	+	+	+	+	+

^a^No enterotoxin genes

^b^No leukotoxin genes

^c^No epidermin genes

**Figure 5:**
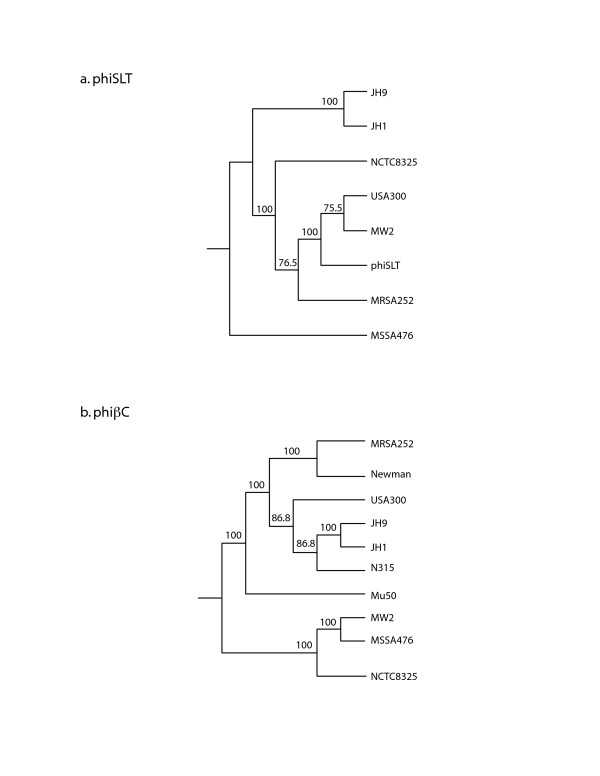
Boot-strapped phylogenetic trees of the prophages found in USA300-HOU-MR: a) phiSLT phage and b) phiβC family phage. The numbers on the branches indicate the number of times the sets co-partitioned after 100 iterations.

USA300-HOU-MR carries two complete prophage sequences, φSLT-USA300 and φβC-USA300. Prophage remnants also occur within pathogenicity island SaPI5. The φSLT-USA300 sequence is 98% identical to the 46139 bp φSLT sequence [28], excepting gaps greater than 500 bp. These gaps represent regions in USA300 encoding a helicase and virulence-associated protein E, two DNA polymerases, and two prophage regulatory proteins. The prophage carries the Panton-Valentine leukocidin genes *lukS-PV *and *lukF-PV*. By PHYLIP analysis, the φSLT-USA300 sequence is most similar to the prophages in MW2 and clinical strain A980470 (phiSLT) [28] (Figure 5a). As observed for φSLT-A980470 and φ-PVL, the φSLT-USA300 attachment site lies within a conserved hypothetical protein gene [28,29]. The second prophage, φβC-USA300, is very similar to β-hemolysin-converting prophages identified in JH1, JH9, MRSA252, MSSA476, Mu50, MW2, N315, Newman, and NCTC8325 (Figure 5b). It is most similar to those found in JH1, JH9 and N315. In USA300, this prophage encodes staphylokinase, chemotaxis inhibitory protein and staphylococcal complement inhibitor; the order of these genes places it in immune evasion cluster (IEC) type B [30]. Unlike COL, USA300-HOU-MR lacks the L54α-like bacteriophage. As a result, the glycerol ester hydrolase, or *geh *lipase gene, which is disrupted in strains carrying the L54α-like prophage, is intact and presumed to be expressed.

### Plasmids

**Figure 6:**
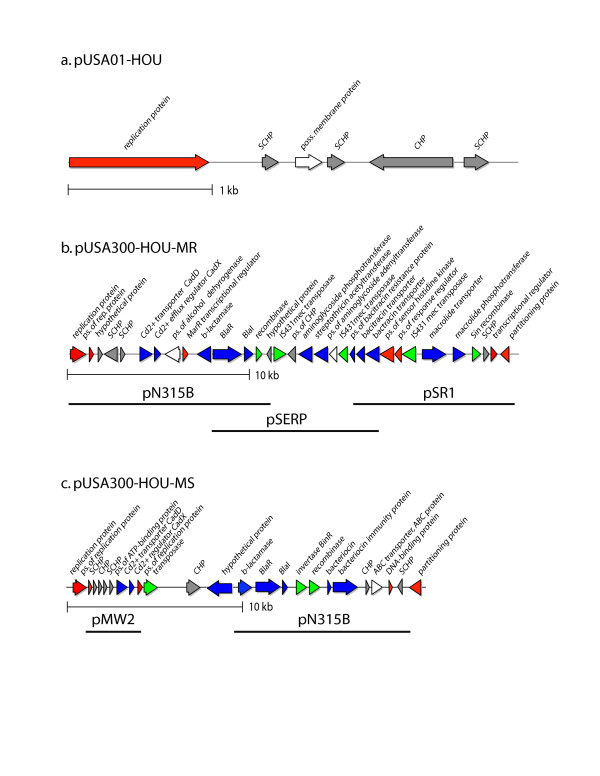
Linear representations of circular plasmids observed in USA300-HOU-MR (a, b) and USA300-MS (c). Replication and regulatory genes are colored red, recombination/transposition genes are green, antibiotic/heavy metal/bacteriocin resistance genes are blue, other genes are white and hypothetical, conserved hypothetical (CHP) and staphylococcal conserved hypotheticals (SCHP) are colored grey. Horizontal bars indicate the regions of pUSA300-HOU-MR and pUSA300-HOU-MS having homology with other sequenced staphylococcal plasmids.

Two plasmids were detected in USA300-HOU-MR (Figure 6a and 6b). The 3.1 kb plasmid, pUSA01-HOU, is identical to plasmid pUSA01 in USA300-FPR3757. It encodes a replication protein plus several conserved hypothetical proteins. The second larger plasmid, pUSA300-HOU-MR, appears to be a mosaic of parts of three plasmids: pN315 from *S. aureus *N315; pSR1 from *S. aureus *strain 01A103; and pSERP from *S. epidermidis *RP62A. The plasmid carries two recombinase genes and three IS*431 *transposase genes that may have played a role in fusion of the three elements. The replication region appears to be derived from pNF315B. pUSA300-HOU-MRSA encodes resistance to beta-lactam, macrolide, aminoglycoside, streptothricin (nourseothricin) and bacitracin antibiotics, consistent with the resistance pattern observed for USA300-HOU-MR (Table 3), with the exception of nourseothricin. The streptothricin acetyltransferase and 3',5'-aminoglycoside transferase genes are adjacent and map between two IS*431 *transposase genes; long inverted repeats (*ca*. 700 bp) flank these genes. The repeats are most similar to the inverted repeats found in IS*257 *from *S. epidermidis *strain SK76 (Genbank SEU40386) though similar repeats are found on a number of plasmids from both *S. aureus *and *S. epidermidis*. Thus, it appears that this region constitutes a compound transposon that could also be part of a larger element carrying the bacitracin transporter genes. A ca. 400 nt repeat (which is a subset of the ca 700 bp repeat) also occurs within the IS*431 *transpose genes at ca. 12.5 and 21 kb on the map. pUSA300-HOU-MR also encodes a cadmium transporter CadD and regulator CadX, and at least three additional predicted transcriptional regulators including BlaI and BlaR.

**Table 3:** Antimicrobial susceptibilities of USA300-HOU-MR and USA300-HOU-MS

**Antibiotic**	**USA300-HOU-MR**		**USA300-HOU-MS**	
	**MIC**	**Phenotype**	**MIC**	**Phenotype**
Bacitracin	>128	R	32	S
Cephalexin	128	R	4	S
Cefazolin	32	R	8	S
Cefotaxime	>128	R	2	S
Ciprofloxacin	0.5	S	0.25	S
Clarithromycin	64	R	<0.125	S
Clindamycin	0.06	S	0.06	S
Doxycycline	<0.125	S	<0.125	S
Erythromycin	64	R	<0.125	S
Gentamicin	1	S	1	S
Kanamycin	>128	R	4	S
Minocycline	<0.125	S	<0.125	S
Nourseothricin	8	S	4	S
Oxacillin	128	R	1	S
Penicillin	32	R	4	R
Rifampin	<0.004	S	<0.004	S
Tetracycline	<0.125	S	<0.125	S
Trimethoprim-Sulfamethoxazole	<0.03/0.06	S	0.06/0.125	S
Vancomycin	1	S	1	S

MIC, minimum inhibitory concentration, μg/ml

R, resistant; S, susceptible

### Comparison of USA300-HOU-MR and USA300-FPR3757

We identified 14 insertions/deletions (indels) of greater than four bp between USA300- FPR3757 and USA300-HOU-MR (Table 4). The most significant difference between the two strains is the location of a 13.4 kb insertion. In USA300-FPR3757, this region is inserted at nt 1,630,721 between a biotin carboxylase carrier protein gene, *accB*, and the elongation factor P gene *efp*. In USA300-HOU-MR, this same 13 kb segment of DNA is inserted at nt 680,375 within the *snoA *gene at the beginning of the staphylococcal *nuo*-like operon, which encodes an NADH dehydrogenase [31]. The 13 kb segment is also found in COL, at the same location as in USA300-FPR3757, and in MRSA252 between two bacteriophage protein genes. There is no homology flanking the insertions sites for this element and there are no obvious recombination sequences or repeats within the segment. The first gene in the segment encodes a frameshift of an IS*SA1 *transposase. The frameshift occurs in a poly adenine tract so sequence variation through slipped-strand mispairing is possible. Six of the proteins encoded by the 13 kb segment may be non-classically secreted proteins. Also encoded are a possible lipoprotein, a peptidoglycan hydrolase, a cell division protein, a GTP-binding protein, a replication protein and a possible actin-binding protein.

**Table 4:** Insertions and deletions between USA300-HOU-MR and FPR3757

**Coordinates (nts)**			
**USA300-HOU-MR**	**FPR3757**	**Length (nts)**	**Comments**
52999	52999–53053 insertion	55	*mec*IV*a *region
128604	128663–128710 insertion	47	IgG binding protein A
556303–556952 insertion	556410	643	rRNA
680918–694274 insertion	680375	13357	plasmid-like sequences
749056	735156–735224 insertion	69	hypothetical protein
855698–855815	841867	118	hypothetical protein
1644669	1630721–1644121 insertion	13401	plasmid-like sequences
1857878–1858036	1857330	159	within repeat
1995735	1995030–1995080 insertion	51	within repeat
2031742	2031088–2031146 insertion	59	within repeats between 2 CHPs^a^
2179534	2178939–2179061 insertion	123	rRNA
2179703	2179232–2179352 insertion	121	rRNA
2295844	2295493–2295615 insertion	123	rRNA
2296014	2295786–2295905 insertion	120	rRNA

^a^CHP, conserved hypothetical protein

An insertion of 159 bp in USA300-HOU-MR but not in USA300-FPR3757 occurs within a staphylococcal conserved hypothetical protein between 1857878 and 1858036. This insertion is a duplication of flanking sequence and maintains the reading frame of the protein. Another difference identified is a 47 bp region in USA300-FPR3757, within the repeated region of the IgG binding protein gene; again, these are absent from USA300-HOU. Additional short deletions in USA300-HOU-MR also occur within intergenic regions. Also of note is a frameshift in the gene encoding an AraC family transcriptional regulator in USA300 (742860–745009). This frameshift is not observed in any of the other sequenced *S. aureus *strains. In addition, we identified a frameshift in the cell wall anchor protein gene (2623054–2626400) not reported in the annotation of FP3757.

**Figure 7:**
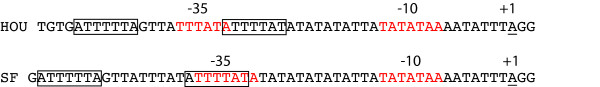
DNA sequence of the *sarU *promoters in USA300-HOU-MR and FPR3757 showing -10 and -35 sequences (red) and SarA consensus binding sites (boxed).

Comparison of the two USA300 sequences revealed 92 single nucleotide polymorphisms (SNPs) and two four bp. deletions (Additional file 2). We also discovered 53 sequencing errors in the FPR357 sequence. Fifty-five of the SNPs map within ORFs, while 18 map within intergenic regions (this is consistent with the fact that 80% of the genome is coding sequence) and 7 map within rRNA sequences. Twenty-one of the changes within reading frames are synonymous, 34 are non-synonymous and 7 caused frameshifts. Two of the SNPs may explain differences in antibiotic susceptibility. One, a T (FPR357) to C (HOU) change in the *gyrA *gene causes a leucine to serine change at amino acid 84. The serine-containing GyrA protein confers resistance to ciprofloxacin (FPR357), while leucine confers the wild-type, susceptible phenotype (HOU, Table 2) [32]. A second SNP presumed to be involved in fluoroquinolone resistance maps within the topoisomerase IV *parC *gene. Here, the USA300-HOU-MR amino acid 80 is the wild-type serine, while USA300-FPR3757 has a tyrosine that confers resistance [33]. Three non-synonymous SNPs occur within the IgG binding protein A. SNPs in intergenic regions could affect gene regulation: a four base deletion at 2,627,226 would place a SarA binding motif closer to the *sarU *promoter and could affect its transcription (Figure 7).

We compared the USA300 SNPs to all other sequenced *S. aureus *strains (Additional file 2). For 39 of the SNPs, the other strains had the USA300-HOU-MR allele, while in 34 the allele was identical to FPR3757. For the remaining SNPs, the other strains showed both alleles, with some being triallelic.

### Comparison of USA300-HOU-MR and of USA300-HOU-MS

We generated a draft sequence of the methicillin susceptible strain, USA300-HOU-MS, using the 454 Life Sciences pyrosequencing technology [34]. This strain is susceptible to all the antibiotics tested except penicillin (Table 1). The sequences were assembled into 326 contigs with a total contig length of 2.8 Mb. We observed 32 SNPs between the HOU-MRSA and MSSA strains (Additional file 1). We also identified two regions of DNA that are found in USA300-HOU-MR but not in USA300-HOU-MS. In contrast, we observed only one region in the MS strain not present in the USA300-HOU-MR strain. This 733 bp contig had a short DNA match (44 bp, 100% identity) to several staphylococcal sequences and a partial protein match (50% length, 38% identity) to a transport accessory protein. The sequences missing from USA300-HOU-MS included the SCC*mec *and ACME regions (34173–88845, Additional file 2). A second region not found in USA300-HOU-MS (680132 to 694219) includes a large number of staphylococcal conserved hypothetical proteins (Additional file 2). Four of the proteins encoded by this region may be secreted (two non-classical). Also encoded are a possible lipoprotein, a peptidoglycan hydrolase, a cell division protein, ATP- and GTP-binding proteins, a replication protein, a possible secreted actin-binding protein, a possible ornithine carbamoyltransferase and a possible histidinol dehydrogenase.

We surveyed other sequenced staphylococcal genomes using TBLASTX for the presence of ORFs that were homologous to proteins encoded by the regions of USA300-HOU-MR that were missing in the MSSA strain. These are shown in Additional file 2. With the exception of a few matches in the MW2 genome, the region from 51299 to 62735 seems to encode proteins unique to USA300-MR. This region also encodes four hypothetical and six conserved hypothetical proteins; several of these, although present, were not annotated in USA300-FPR3757. As previously discussed, this region may encode several secreted proteins. Though the arginine deiminase DNA region is unique to USA300 and to *S. epidermidis *ATCC 12228, genes encoding homologues of the proteins are found elsewhere in the USA300-HOU-MS genome and in most of the complete staphylococcal genomes. Proteins weakly similar (e value > e-10) to the nickel transporter were found in all staphylococcal genomes but this is likely due to generalized matches to other ABC transporter subunits. In contrast, the nickel transporter proteins are most homologous to proteins encoded by *Staphylococcus haemolyticus*. The proteins encoded by the region between 680132 and 694219 are also encoded by the genomes of two nosocomial MRSA strains, COL and MRSA252. These were not found in the CA isolate MW2.

USA300-HOU-MS does not carry plasmid pUSA300-HOU-MR but it does carry a 21 kb plasmid (pUSA300-HOU-MS) that encodes resistance to beta-lactams and to cadmium (Figure 6c). It also carries genes that are predicted to encode a bacteriocin and its associated immunity protein. The region from 9382 to 13575 on pUSA300-HOU-MS is nearly identical (95%) to nts 7644 to 11837 of plasmid pN315B. This corresponds to the region encoding the beta-lactamase gene and its regulators, BlaR and BlaI, and a Tn*552 *invertase and recombinase. In addition, this same region on pUSA300-HOU-MS is 96% identical to nts. 7615–13573 on pUS300-HOU-MR. Thus, pUSA300-HOU-MS also appears to be mosaic. We also identified sequences homologous to pUSA01 on two contigs that were 1212 and 1278 bp in length, respectively. Since pUSA01 has now been sequenced twice, we did not attempt to complete the pUSA01 sequence in the MS strain.

### Comparison with other completely sequenced staphylococcal genomes

**Figure 8:**
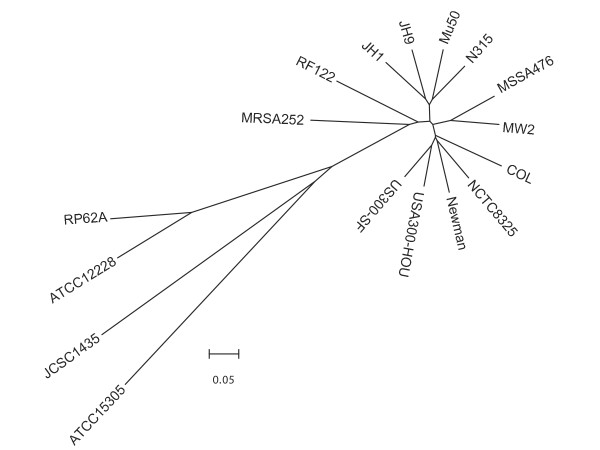
Dendrogram of seventeen fully sequenced genomes generated by Mauve [35] and drawn using MEGA [58]. RP62A and ATCC12228 are *S. epidermidis *strains, JCSC1435 is an *S. haemolyticus *strain, and ATCC15305 is an *S. saprophyticus *strain. All others are *S. aureus *strains. The bar represents coalescent units.

We used Mauve [35] to align the sequences of the seventeen completed sequenced staphylococcal genomes (Figure 8) The alignment revealed that USA300 clusters with COL, NCTC8325, and Newman strains and that there are at least four clades of MRSA circulating. This clustering suggests that not all methicillin resistant S. aureus strains are intimately related further suggesting that different clones have given rise to groups of methicillin strains.

## Discussion

Because of the rapid and continuing increase of the prevalence of CA USA300 strains at TCH, we initiated the sequencing of two typical isolates from pediatric patients. During this time, Diep *et al*. [24] reported the sequence of USA300-FPR3757. USA300-HOU-MR revealed significant differences from USA300-FPR3757. Though USA300-FPR3757 was chosen as a highly resistant isolate, USA300-HOU-MR, a purely community-acquired strain, harbours a large plasmid encoding multiple antibiotic and cadmium resistance. The mosaic composition of plasmid pUSA300-HOU-MR representing three different staphylococcal plasmids points toward significant genetic exchange between staphylococcal isolates and species. Among the numerous antibacterial resistance genes found on the plasmid, the conferred resistance to bacitracin, a common constituent of topical over-the-counter ointments used to treat or prevent cutaneous infections, is most intriguing. In contrast to other MR strains, the cadmium resistance genes were found on a plasmid and not on the chromosome. Genome-wide alignments revealed that not all MRSA are tightly clustered.

Several indels between USA300-HOU-MR and USA300-FPR3757 were identified. The majority of the indels were within repeat regions or rRNAs, though a 13 kb segment was located in different regions of the two genomes. In USA300-FPR3757, the region is inserted between two genes. In USA300-HOU-MR, the segment interrupts an operon (*snoABCDEFG*) that has been implicated in susceptibility to thrombin-induced platelet microbicidal protein 1 (tPMP-1). Bayer *et al*. [31] have shown that interruption of this operon reduces the susceptibility of *S. aureus *to tPMP-1 *in vitro*. Resistance to tPMP-1 may allow US300-HOU-MR to cause more serious disease by evasion of platelet-mediated killing in the blood stream. USA300-FPR3757 has an additional repeat within the gene encoding the IgG binding protein A, so it is possible that IgG binding protein is subject to structural variation. We also identified nine SNPs within this gene. This may suggest that the IgG binding protein undergoes significant variation, possibly in response to the host.

Two prophage were identified, one carrying the PVL genes and the other an hemolysin converting phage carrying the staphylokinase, chemotaxis inhibitory protein and staphylococcal complement inhibitor genes. USA300 lacks a copy of prophage L54a. The GehD lipase, whose gene contains the prophage attachment site, is likely to play a role in colonization events. [36].

SNP analysis revealed 47 non-synonymous, non-conserved differences between the strains. Some of these may affect protein function. Two of these, one in *gyrA *and one in *parC*, are consistent with antibiotic susceptibilities reported in Table 3. The SNP analysis also revealed a number of sequencing errors in the FPR3757 sequence.

To further our understanding of the evolution of the USA300-HOU-MR strain, we created a draft sequence of an MS isolate from TCH. This revealed two regions of the MR strain not found in the MS strain. It is likely that the progenitor MS strain acquired these regions (along with the SCC*mec*IVa and the arginine deiminase region) by recombination to become USA300-HOU-MR. We propose that USA300-HOU-MS first acquired the arginine deiminase region plus the cassette chromosome recombinase genes (*ccrA*B) from a strain similar to *S. epidermidis *ATCC12228. This is based on the observation that the *ccrAB *genes in USA300-HOU-MR are 95–100% identical to those found near the arginine deiminase region in *S. epidermidis *ATCC12228. The SCC*mec *was likely acquired in a separate event from another MRSA, or from a MR-*S. epidermidis strain*, via *ccrAB *recombination within the repeats flanking the arginine deiminase region [24]. US300-HOU-MS lacks a probable nickel ABC transporter that is linked to ACME in USA300-MR. It is possible that increased intracellular stores of nickel in USA300-HOU-MR could contribute to virulence by enhancing the activity of the nickel-dependent urease. [37]. The MS strain also lacks a copy of the P-ATPase copper transporter gene; such transporters have been shown to be involved in virulence in *Listeria monocytogenes *[38]. Another potential virulence factor revealed by comparison of the MR and MS strains is a possible secreted actin binding protein.

We used a relatively new predictive tool, SecretomeP2.0, to identify non-classically secreted proteins [39] since it is not possible to predict the function of most of these proteins by BLAST analysis. We found 406 high scoring genes predicted to encode non-classically secreted proteins in USA300-HOU-MR (Additional file 3). If some of these are indeed secreted then they may be candidate virulence factors for future study.

## Conclusion

Comparative analyses have revealed that USA300-MRSA is highly similar to some MRSA strains suggesting that the differences in pathogenesis are due to subtle changes rather than to large-scale acquisition of virulence factor genes. Significantly, however, is the difference in plasmid content and antibiotic susceptibility profiles between the two USA300 isolates. The presence of a large multidrug resistance plasmid in a community-acquired isolate is unexpected. The combination of Sanger sequencing and 454 data allowed us to economically derive the complete sequence of USA300-HOU-MR and MS. Future sequencing of additional USA300 isolates will further characterize the genotypic variation in this clone. With exception of the antibiotic susceptibility profiles, the phenotypic results of the genotypic variation have yet to be discovered.

## Methods

### USA300 isolates

Clinical isolate TCH1516 (USA300-HOU-MR) was obtained from an adolescent patient with severe sepsis syndrome [15]. The strain was representative of the predominant *Sma *I pulsed field gel electrophoresis (PFGE) pattern (Figure 2) observed in our clonal studies on CA-MRSA at TCH [9] and is indistinguishable from the CDC type strain USA300.0114 obtained from the Network on Antimicrobial Resistance in *Staphylococcus aureus *[40]. The strain is sequence type 8 (ST8). Clinical isolate TCH959 (USA300-HOU-MS) was obtained from a 12 year old white female with a buttock abscess. The PFGE profile of the MSSA strain differed from USA300.0114 by two fragments. USA300-HOU-MS lacked a fragment of ca. 230 kb, which is present in the USA300-HOU-MR and the MS pattern included a 180 kb fragment absent in MR. Additionally, the *ca*. 300 kb fragment was slightly larger in the MS strain. FPR3757 was kindly provided by Francois Perdreau-Remington (University of California, San Francisco).

### Antimicrobial susceptibilities

Antimicrobial susceptibilities were obtained by microbroth dilution assays using Clinical Laboratory Standards Institute (CLSI) guidelines [41] (Table 3). Unlike the other sequenced USA300 isolate [24], USA300-HOU is susceptible to clindamycin, tetracycline, ciprofloxacin and mupirocin.

### DNA sequencing and genome assembly

Genomic DNA was purified from CsCl gradients [42] and DNA sequencing was performed by Sanger dideoxy whole genome shotgun (WGS) and by the 454 Life Sciences pyrosequencing strategies [34]. Genomic and plasmid DNA from USA300-HOU-MR was sheared to a size of 2 kb by nebulization, and cloned into a derivative of pUC18 [43]. The clones were used for WGS DNA sequencing to 8× coverage by using dye terminator chemistry, data were collected on ABI 3730 sequencers, and reads were assembled using the ATLAS assembler [44]. This sequence was supplemented with contigs assembled from shorter reads generated on a 454 Life Sciences GS20 sequencer to 15× coverage. This combined assembly was refined by sequencing PCR products to fill in gaps and to resolve ambiguous regions. DNA from USA300-HOU-MS was sequenced by the 454 method to 17× coverage and the sequences assembled using the 454 Newbler assembler. The complete sequences have been deposited at DDBJ/EMBL/GenBank under the following the accession numbers: CP000730 (USA300-HOU-MR); CP000731 (pUSA300-HOU-MR); CP000732 (pUSA300-HOU-MS). The USA300-HOU-MS has been deposited under the project accession AASB00000000. The version described in this manuscript is the first version, AASB01000000.

Glimmer [45] and GeneMark [46] were used independently to predict open reading frames (ORFs). Predicted proteins were compared to the nr database using BLASTP. Visualization of gene predictions was performed using the Genboree system [47] and the CONAN database [48]. Two individuals independently annotated each ORF and annotations were reconciled and converted to single entries within the database. Proteins lacking a homolog in the nr database were called hypothetical proteins, those with a homolog of no known function were called conserved hypothetical proteins and of these, if homologs were only in other staphylococci they were called staphylococcal conserved hypothetical proteins.

### Sequence analysis

DNA comparisons were performed with BLASTN and BLASTZ. Protein sequences were analyzed by BLASTP and BLASTX and by the use of the predictive tools InterProScan [49], PFP [50], PSORTb [51], Helix-Turn-Helix Predictor [52], MEROPs [53], and the Tcdb Transport Classification database [54]. Non-classically secreted proteins (those lacking signal peptides) were predicted using SecretomeP2.0 [39]. Riboswitches and non-coding small RNAs were identified using BLASTN, the Riboswitch finder [55] and Riboswitch Explorer [56]. ClustalW [57] and Mauve [35] were used for multiple sequence alignments and PHLYIP bootstrapping and tree generation (evolution.genetics.washington.edu/phylip.html). Single nucleotide polymorphisms (SNPs) between USA300-MRSA-HOU and FPR3757 were verified by resequencing.

## Authors' contributions

SKH performed much of the post-annotation analysis and wrote the draft of the manuscript. SKH, KGH, EOM, RMF, YL, OI, SY, JP, MT, AU, GEF annotated the genome. XQ and HJ contributed bioinformatics support. YS, TMW and AMC performed the SNP verification. DMM, LH, YD, SD, PRB, CJB, HHD, ACH, MH, CLK, SLL, WL, LVN, QW and JZ composed the sequencing and finishing team. KGH, RMF, EOM, SLK and GMW assisted critical review of the manuscript. All authors read and approved the final manuscript.

## Supplementary Material

Additional file 1 DNA regions present in USA300-HOU-MR but missing in USA300-HOU-MS and their presence in other sequenced staphylococcal genomes. Click here for file

Additional file 2 Single nucleotide polymorphisms and short insertions/deletions between USA300-MR, USA300-MS and FPR3757. Alleles in other sequenced *S. aureus *strains are also listed. Click here for file

Additional file 3 Predicted non-classically secreted proteins. Click here for file
