# Supplementary material for: Subtle genetic changes enhance virulence of methicillin resistant and sensitive Staphylococcus aureus
Source: BMC Microbiol. 2007 Nov 6;7:99. doi: 10.1186/1471-2180-7-99 (PMC2222628; doi:10.1186/1471-2180-7-99)
Supplement: Additional file 1 — DNA regions present in USA300-HOU-MR but missing in USA300-HOU-MS and their presence in other sequenced staphylococcal genomes. [file 1471-2180-7-99-S1.doc]

Supplemental Table 1 - DNA regions present in USA300-HOU-MR but missing in USA300-HOU-MS and their presence in other completely sequenced staphylococcal genomes.

| **START** | **STOP** | **STRAND** | **DEFINITION IN HOU** | **USA300 MRSA** | **USA300 MSSA** | **Newman** | **NCTC**  **8325** | **COL** | **MW2** | **MSSA 476** | **N315** | **Mu50** | **JH1** | **JH9** | **RF122** | **MRSA 252** | **RP62A** | **ATCC 12228** | **JCSC 1435** | **ATCC**  **15305** |  |
| --- | --- | --- | --- | --- | --- | --- | --- | --- | --- | --- | --- | --- | --- | --- | --- | --- | --- | --- | --- | --- | --- |
|  |  |  | **34474-91900** |  |  |  |  |  |  |  |  |  |  |  |  |  |  |  |  |  |  |
| 34474 | 35769 | + | conserved hypothetical protein, possible transcriptional regulator | + | - | - | - | + | + | + | + | + | + | + | - | + | + | - | - | - |  |
| 35877 | 36551 | - | IS431mec transposase | + | - | - | - | + | + | - | + | + | + | + | + | + | + | + | + | + |  |
| 37074 | 37304 | + | staphylococcal conserved hypothetical protein | + | - | - | - | + | + | - | + | + | - | - | - | + | + | - | + | - |  |
| 37813 | 38556 | + | glycerophosphodiester phosphodiesterase | + | +/- | - | +/- | + | + | +/- | + | + | + | + | +/- | + | + | +/- | + | +/- |  |
| 38653 | 39081 | + | probable acyl dehydratase MaoC | + | - | - | - | + | + | - | + | + | + | + | - | + | + | - | + | - |  |
| 39127 | 41133 | - | penicillin binding protein 2 prime | + | - | - | - | + | + | - | + | + | + | + | - | + | + | - | + | - |  |
| 41305 | 42219 | + | methicillin resistance regulatory protein MecR1 | + | - | - | - | + | + | - | + | + | + | + | - | + | + | +/- | - | - |  |
| 42143 | 42451 | - | possible type I site-specific deoxyribonuclease restriction subunit | + | - | - | - | + | + | - | - | - | - | - | - | - | - | - | - | - |  |
| 42442 | 43965 | - | IS*1272* transposase | + | - | - | + | + | + | - | + | + | + | + | - | + | - | + | + | - |  |
| 44101 | 44610 | - | conserved hypothetical protein, possible RNA polymerase | + | - | - | - | + | + | + | + | + | + | + | - | + | + | + | + | +/- |  |
| 44622 | 44933 | - | staphylococcal conserved hypothetical protein | + | - | - | - | + | + | + | + | + | + | + | - | + | + | + | + | +/- |  |
| 44935 | 45027 | - | staphylococcal conserved hypothetical protein, probable extracellular protein | + | - | - | - | +/- | + | + | - | - | - | - | - | - | - | +/- | +/- | - |  |
| 45020 | 45370 | - | staphylococcal conserved hypothetical protein, possible tRNA ligase | + | - | - | - | + | + | + | + | + | + | + | - | + | + | + | +/- | +/- |  |
| 45892 | 47520 | - | cassette chromosome recombinase B | + | - | - | - | +/- | + | +/- | + | + | + | + | - | + | + | + | +/- | +/- |  |
| 47542 | 48891 | - | cassette chromosome recombinase A | + | - | - | - | + | + | + | + | + | + | + | - | + | + | + | - | +/- |  |
| 49125 | 50912 | - | conserved hypothetical protein, possible helicase | + | - | - | - | + | + | + | + | + | + | + | - | + | + | + | - | +/- |  |
| 50912 | 51202 | - | staphylococcal conserved hypothetical protein, possible primase, possible non-classically secreted protein | + | - | - | - | +/- | + | +/- | +/- | +/- | - | - | - | +/- | +/- | +/- | - | - |  |
| 51299 | 52390 | + | staphylococcal conserved hypothetical protein | + | - | - | - | - | + | - | - | - | - | - | - | - | - | - | - | - |  |
| 53063 | 54553 | + | possible transcriptional regulator, possible non-classically secreted protein | + | - | - | - | - | + | - | - | - | - | - | - | - | - | - | - | - |  |
| 55177 | 56181 | - | probable beta-lactamase | + | +/- | - | - | + | + | - | - | - | + | + | - | - | + | + | - | - |  |
| 56300 | 56437 | + | hypothetical protein, probable extracellular protein | + | - | - | - | - | + | - | - | - | - | - | - | - | - | - | - | - |  |
| 56934 | 57554 | + | staphylococcal conserved hypothetical membrane protein | + | - | - | - | - | + | - | - | - | - | - | - | - | - | - | - | - |  |
| 58471 | 58641 | + | hypothetical protein, possible RNA polymerase | + | - | - | - | - | - | - | - | - | - | - | - | - | - | - | - | - |  |
| 59043 | 60008 | + | conserved hypothetical protein, possible endonuclease | + | - | - | - | - | + | - | - | - | - | - | - | - | - | - | - | - |  |
| 60105 | 60296 | + | hypothetical protein | + | - | - | - | - | - | - | - | - | - | - | - | - | - | - | - | - |  |
| 60388 | 60717 | + | staphylococcal conserved hypothetical protein | + | - | - | - | - | - | - | - | - | - | - | - | - | - | - | +/- | - |  |
| 60794 | 61132 | - | conserved hypothetical membrane protein, possible non-classically secreted protein | + | - | - | - | - | - | - | - | - | - | - | - | - | - | - | - | - |  |
| 61393 | 61560 | - | hypothetical protein, probable extracellular protein | + | - | - | - | - | - | - | - | - | - | - | - | - | - | - | - | - |  |
| 61724 | 62533 | + | integrase | + | - | - | - | - | - | - | + | + | + | + | - | + | + | + | - | - |  |
| 62553 | 62735 | - | staphylococcal conserved hypothetical protein | + | - | - | - | - | - | +/- | - | - | - | - | - | - | - | - | - | - |  |
| 63046 | 63543 | - | diamine N-acetyltransferase | + | - | - | - | - | - | - | - | - | - | - | - | - | + | + | + | + |  |
| 63731 | 63859 | + | staphylococcal conserved hypothetical protein | + | - | - | - | - | - | +/- | - | - | - | - | - | - | +/- | +/- | +/- | - |  |
| 64071 | 65168 | - | alcohol dehydrogenase | + | +/- | - | +/- | +/- | +/- | +/- | +/- | +/- | - | - | - | +/- | + | + | + | + |  |
| 65423 | 65647 | - | staphylococcal conserved hypothetical protein, possible tRNA ligase | + | - | - | +/- | - | - | + | - | - | - | - | +/- | - | + | + | + | - |  |
| 65661 | 66164 | - | conserved hypothetical protein, possible RNA polymerase | + | - | - | - | + | + | + | + | + | + | + | - | + | + | + | + | +/- |  |
| 66180 | 66506 | - | staphylococcal conserved hypothetical protein | + | - | - | - | + | + | + | + | + | + | + | - | + | + | + | + | - |  |
| 66493 | 66585 | - | staphylococcal conserved hypothetical protein | + | - | - | - | + | + | + | + | + | +/- | +/- | - | + | + | + | + | - |  |
| 66578 | 66928 | - | staphylococcal conserved hypothetical protein | + | - | - | - | + | + | + | + | + | + | + | - | + | + | + | +/- | +/- |  |
| 67885 | 68736 | + | transposase | + | - | - | - | - | +/- | +/- | + | + | + | + | - | + | + | + | - | - |  |
| 68836 | 69765 | - | carbamate kinase | + | + | + | + | + | + | + | + | + | + | + | + | + | + | + | + | - |  |
| 69785 | 70783 | - | ornithine carbamoyltransferase | + | + | + | + | + | + | + | + | + | + | + | + | + | + | + | + | +/- |  |
| 70821 | 71510 | - | Crp family transcriptional regulator | + | +/- | +/- | + | +/- | - | +/- | +/- | +/- | +/- | +/- | +/- | +/- | +/- | + | +/- | - |  |
| 71552 | 72973 | - | arginine/ornithine APC family transporter, possible non-classically secreted protein | + | + | +/- | + | +/- | +/- | +/- | +/- | +/- | +/- | +/- | +/- | +/- | + | + | + | - |  |
| 73059 | 74294 | - | arginine deiminase | + | +/- | + | + | + | + | + | + | + | + | + | + | + | + | + | + | - |  |
| 74563 | 75009 | - | arginine repressor | + | +/- | +/- | +/- | +/- | +/- | +/- | +/- | - | +/- | +/- | +/- | +/- | +/- | + | +/- | - |  |
| 75511 | 75942 | + | universal stress protein | + | - | - | - | - | - | - | - | - | - | - | - | - | + | + | - | - |  |
| 77076 | 77891 | + | lysophospholipase | + | - | - | - | - | - | - | - | - | - | - | - | - | - | - | - | + |  |
| 78378 | 79391 | + | probable transposase | + | - | - | - | - | - | - | - | - | - | - | + | - | + | + | - | - |  |
| 79651 | 80343 | + | possible S-adenosyl-L-methionine-dependent methyltransferase | + | - | - | - | - | - | - | - | - | - | - | - | - | - | - | + | - |  |
| 80340 | 81893 | + | nickel (Ni2+)/peptide ABC superfamily ATP binding cassette transporter, binding protein | + | - | +/- | +/- | +/- | +/- | +/- | +/- | +/- | +/- | +/- | +/- | +/- | +/- | +/- | + | - |  |
| 81896 | 82852 | + | nickel (Ni2+)/peptide ABC superfamily ATP binding cassette transporter, membrane protein | + | - | +/- | +/- | +/- | +/- | +/- | +/- | +/- | +/- | +/- | +/- | +/- | +/- | +/- | + | +/- |  |
| 82852 | 83619 | + | nickel (Ni2+)/peptide ABC superfamily ATP binding cassette transporter, membrane protein | + | - | - | - | - | - | - | - | - | - | - | - | - | - | - | + | - |  |
| 83586 | 84353 | + | nickel (Ni2+)/peptide ABC superfamily ATP binding cassette transporter, ABC protein | + | - | - | - | - | - | - | - | - | - | - | - | - | - | - | + | - |  |
| 84346 | 84981 | + | nickel (Ni2+)/peptide ABC superfamily ATP binding cassette transporter, ABC protein | + | +/- | - | - | - | - | - | - | - | - | - | - | - | - | - | + | - |  |
| 86040 | 88064 | - | P-ATPase superfamily P-type ATPase copper (Cu2+) transporter | + | - | +/- | +/- | +/- | +/- | +/- | +/- | +/- | +/- | +/- | +/- | + | + | + | + | +/- |  |
| 88082 | 88627 | + | possible lipoprotein | + | - | - | - | - | - | - | - | - | - | - | - | - | + | + | + | - |  |
| 88819 | 88956 | - | staphylococcal conserved hypothetical protein, possible transposase | + | + | + | + | + | - | - | + | + | + | + | - | - | - | - | - | - |  |
| 91701 | 91799 | - | staphylococcal conserved hypothetical protein | + | - | + | + | + | + | + | + | + | - | - | + | - | - | - | - | - |  |
| 91900 | 92655 | - | conserved hypothetical membrane protein | + | - | + | + | + | + | + | + | + | + | + | + | - | - | - | - | - |  |
|  |  |  | **680132-695295** |  |  |  |  |  |  |  |  |  |  |  |  |  |  |  |  |  |  |
| 680132 | 680281 | - | staphylococcal conserved hypothetical protein, possible tRNA methyltransferase | + | - | + | + | + | + | + | + | + | + | + | + | + | - | - | - | - |  |
| 682267 | 682623 | - | probable lipoprotein | + | - | - | - | - | - | - | - | - | - | - | - | + | - | - | - | - |  |
| 682679 | 683269 | - | staphylococcal conserved hypothetical protein, possible non-classically secreted protein | + | - | - | - | + | - | - | - | - | - | - | - | + | - | - | - | - |  |
| 683276 | 684322 | - | possible C51 family CHAP domain peptidoglycan hydrolase | + | - | - | - | + | - | - | - | - | - | - | - | + | - | +/- | - | - |  |
| 684312 | 686159 | - | conserved hypothetical membrane protein, possible secreted ATP-binding protein | + | - | - | - | + | - | - | - | +/- | - | - | - | + | - | - | - | - |  |
| 686164 | 687522 | - | FtsK/SpoIIIE family cell division protein | + | - | - | - | + | - | - | - | +/- | - | - | - | + | - | - | - | - |  |
| 687576 | 690071 | - | conserved hypothetical membrane protein, possible GTP-binding translational factor | + | - | - | - | + | - | - | - | +/- | - | - | - | + | - | - | - | - |  |
| 690106 | 690489 | - | staphylococcal conserved hypothetical membrane protein, possible non-classically secreted protein | + | - | - | - | + | - | - | - | - | - | - | - | + | - | - | - | - |  |
| 690501 | 690761 | - | staphylococcal conserved hypothetical membrane protein | + | - | - | - | + | - | - | - | - | - | - | - | + | - | - | - | - |  |
| 690766 | 691821 | - | conserved hypothetical protein, possible secreted actin binding protein | + | - | - | - | + | - | - | - | - | - | - | - | + | - | - | - | - |  |
| 691882 | 692973 | - | possible replication protein, possible protein kinase | + | - | - | - | + | - | - | - | +/- | - | - | - | + | - | - | - | - |  |
| 693148 | 693450 | - | staphylococcal conserved hypothetical protein | + | - | - | - | + | - | - | - | - | - | - | - | + | - | - | - | - |  |
| 693464 | 693784 | - | staphylococcal conserved hypothetical protein, possible ornithine carbamoyltransferase | + | - | - | - | + | - | - | - | - | - | - | - | + | - | - | - | - |  |
| 693935 | 694219 | - | staphylococcal conserved hypothetical protein, possible histidinol dehydrogenase | + | - | - | - | + | - | - | - | - | - | - | - | + | - | - | - | - |  |

Comparisons were made using BLASTX and TBLASTX: + = >80% identity; +/-= expect <e-10, over > 90% of query length; - = expect > e -10 or incomplete
