# Supplementary material for: Subtle genetic changes enhance virulence of methicillin resistant and sensitive Staphylococcus aureus
Source: BMC Microbiol. 2007 Nov 6;7:99. doi: 10.1186/1471-2180-7-99 (PMC2222628; doi:10.1186/1471-2180-7-99)
Supplement: Additional file 2 — Single nucleotide polymorphisms and short insertions/deletions between USA300-MR, USA300-MS and FPR3757. Alleles in other sequenced S. aureus strains are also listed. [file 1471-2180-7-99-S2.doc]

**Additional l Table 2 - Single nucleotide polymorphisms and short insertions/deletions between USA300-MR, USA300-MS and FPR3757. Alleles in other *S. aureus* strains are also listed.**

| **Position** | **HOU -MR** | **HOU -MS** | **FPR 3757*** | **Gene or Definition** | **Change  (HOU vs FPR3757)** | **COL** | **MSSA476** | **NCTC8325** | **MW2** | **MRSA252** | **Mu50** | **N315** | **RF122** | **JH1** | **JH9** | **Newman** |
| --- | --- | --- | --- | --- | --- | --- | --- | --- | --- | --- | --- | --- | --- | --- | --- | --- |
| **Single nucleotide polymorphisms (SNPS)** | | | | | |  |  |  |  |  |  |  |  |  |  |  |
| 317 | G | A | A | IGR upstream of *dnaA* | | A | A | A | A | A | A | A | A | A | A | A |
| 5010 | G | G | C | *recF* | GCT -> CCT; A -> P | G | G | G | G | G | G | G | G | G | G | G |
| 7282 | C | C | T | *gyrA* | TCA -> TTA; S -> L | C | C | C | C | T | T | C | C | T | T | C |
| 60970 | A | X | G | CH membrane protein | TTT -> CTT; F -> L | X | X | X | X | X | X | X | X | X | X | X |
| 62543 | A | X | - | IGR upstream of SCHP | | X | X | X | X | - | - | - | X | - | - | X |
| 90261 | - | - | TATA | SCHP | frameshift in HOU | TA | X | TA | X | X | TA | TA | X | TA | TA | TA |
| 128502 | G | G | A | IgG binding protein A | GAC -> GAT; D -> D | A | G | A | G | X | G | G | G | X | X | G |
| 128511 | G | G | A | IgG binding protein A | GGC -> GGT; D -> D | A | T | A | T | X | G | G | T | X | X | G |
| 128517 | C | C | T | IgG binding protein A | AAG -> AAA; K -> K | T | T | T | T | X | T | T | T | X | X | G |
| 128520 | G | G | T | IgG binding protein A | AAC -> AAA; N -> K | T | G | T | G | X | G | G | G | X | X | C |
| 128548 | T | T | C | IgG binding protein A | AAC-> GGC; N -> G | C | C | C | C | X | C | C | C | X | X | G |
| 128549 | T | T | C | IgG binding protein A | AAC-> GGC; N -> G | C | G | C | G | X | C | C | G | X | X | T |
| 128571 | A | A | G | IgG binding protein A | AAT -> AAC; N -> N | G | G | G | G | X | G | G | G | X | X | A |
| 128583 | G | G | A | IgG binding protein A | GGC -> GGT; G -> G | A | A | A | A | X | A | A | A | X | X | G |
| 128595 | A | A | G | IgG binding protein A | AAT -> AAC; N -> N | G | G | G | G | X | G | G | G | X | X | A |
| 180441 | T | C | C | *cap5G* | TAA -> CAA; stop -> Q | C | C | C | C | C | C | C | C | C | C | C |
| 182640 | G | G | T | *cap5I* | GTG -> TTG; V -> L | G | X | G | X | X | G | G | X | G | G | G |
| 199059 | A | - | - | SCHP | frameshift in HOU | - | - | - | - | - | - | - | - | - | - | - |
| 209079 | C | T | T | non-ribosomal peptide synthetase | CTT -> TTT; L -> F | T | T | T | T | T | T | T | T | T | T | T |
| 240253 | A | A | T | lipoprotein | AAA -> AAT; K -> N | A | A | A | A | A | A | A | A | A | A | A |
| 260252 | T | T | C | IGR upstream formate acetyltransferase | | T | T | T | T | T | T | T | T | T | T | T |
| 270405 | C | C | T | 3-hydroxyacyl-CoA dehydrogenase | GCT -> ACT; A -> T | C | C | C | C | C | C | C | C | C | C | C |
| 292633 | C | C | A | sorbitol dehydrogenase | CCG ->ACG; P -> T | C | C | C | C | C | C | C | C | C | C | C |
| 375952 | T | T | C | flavin dehydrogenase | AAC -> GAC; N -> D | T | T | T | T | T | T | T | T | T | T | T |
| 376126 | G | G | T | flavin dehydrogenase | CGT -> AGT; R -> S | G | G | G | G | G | G | G | G | G | G | G |
| 395071 | T | A | A | IGR upstream of acetyltransferase | | A | A | A | A | A | A | A | A | A | A | A |
| 428968 | T | T | C | alkyl hydroperoxide reductase | GAC -> GGC; D -> G | T | T | T | T | T | T | T | T | T | T | T |
| 467444 | T | T | A | tandem lipoprotein | GAT -> GAA; D -> E | T | T | T | T | T | T | T | T | T | T | T |
| 512471 | C | T | T | 16S rRNA |  | C | C | C | C | C | C | C | C | T | T | T |
| 525516 | - | - | A | IGR upstream of *metS* | | - | - | - | - | - | - | - | - | - | - | - |
| 556299 | C | C | T | 5S rRNA |  | X | C | C | C | C | C | C | C | C | C | C |
| 558661 | X | X | T | 16S rRNA |  | C | C | C | C | C | C | C | C | C | C | C |
| 576643 | A | G | G | IGR upstream of serine O-acetyltransferase | | G | G | G | G | G | G | G | G | G | G | G |
| 576847 | A | C | C | IGR upstream of acetyltransferase | | C | C | C | C | C | C | C | C | C | C | C |
| 738541 | T | C | C | PiT phosphate transporter | TAT -> CAT; Y -> H | C | C | C | C | C | C | C | C | C | C | C |
| 749005 | A | T | T | IGR upstream of MFS transporter |  | T | T | T | T | A | A | A | A | T | T | A |
| 749012 | C | T | T | IGR upstream of MFS transporter |  | T | T | T | T | C | C | C | C | C | C | C |
| 783684 | C | T | T | SCHP | CTG -> CTA; L->L | C | C | C | C | C | C | C | C | C | C | C |
| 850938 | T | G | G | CHP | GAT -> GAG; D -> E | T | T | T | T | T | T | T | T | T | T | T |
| 855682 | T | C | C | SCHP | TCC -> CGC; S-> R | T | T | T | T | T | T | T | T | T | T | T |
| 855683 | C | G | G | SCHP | TCC -> CGC; S -> R | C | G | G | G | G | G | G | G | G | G | G |
| 900428 | A | G | G | SCHP | CAA -> CGA; Q -> R | X | X | X | X | G | X | X | G | X | X | X |
| 920455 | C | C | A | IGR upstream of CHP |  | C | C | C | C | C | C | C | C | C | C | C |
| 987659 | - | - | A | SCHP | frameshift in FPR3757 | - | - | - | - | - | - | - | - | - | - | - |
| 1088762 | A | A | G | SCHP | AAT -> GAT; N -> D | A | A | A | A | A | A | A | A | A | A | A |
| 1094736 | G | A | A | IGR upstream of glutaredoxin and  cytochrome oxidase | | A | A | A | A | A | A | A | A | A | A | A |
| 1163297 | A | C | C | CHP | GAT -> GCT; D ->A | C | C | C | C | C | C | C | C | C | C | C |
| 1229860 | C | C | T | cytosine-C5-methylase | CAT -> TAT; H -> Y | C | C | C | C | C | C | C | C | C | C | C |
| 1241653 | G | A | A | *recG* | GGT -> GAT; G -> D | A | A | A | A | A | A | A | A | A | A | A |
| 1283489 | T | T | A | *polC* | ATT -> ATA; I -> I | T | T | T | T | T | T | T | T | T | T | T |
| 1300933 | G | G | A | *ftsK* | AGA -> AAA; R -> K | G | G | G | G | G | G | G | G | G | G | G |
| 1309444 | G | G | A | *cinA* | GCG -> GCA; A -> A | G | G | G | G | G | G | G | G | G | G | G |
| 1346362 | A | G | G | threonine aldolase | ACT -> ACC; T -> T | A | G | A | G | G | G | G | G | G | G | G |
| 1377154 | T | T | A | *sbcC* | TTT -> ATT; F -> I | T | T | T | T | T | T | T | T | T | T | T |
| 1388352 | C | C | A | *parC* | TCC -> TAC; S -> Y | C | C | C | C | T | T | C | C | A | A | C |
| 1547259 | T | T | A | IGR upstream of S1 |  | T | T | T | T | X | X | X | X | A | A | T |
| 1582892 | T | T | C | phiSLT portal protein | CCA -> CCG; P -> P | T | T | T | T | T | X | X | X | T | T | X |
| 1608328 | C | T | T | CHP | ACG -> ACA T -> T | T | T | T | T | T | T | T | C | C | C | C |
| 1694285 | C | C | T | coproporphyrinogen III oxidase | GCA -> ACA; A -> T | C | C | C | C | C | C | C | C | C | C | C |
| 1765610 | C | C | A | *valS* | CCG -> CCT; P -> P | C | C | C | C | C | C | C | C | C | C | C |
| 1847565 | T | T | - | IGR upstream of cell wall anchored protein |  | A | T | A | T | A | A | A | A | T | T | T |
| 1857839 | T | T | C | SCHP | AAA -> AAG; K -> K | T | T | T | T | C | T | T | C | C | C | T |
| 1857867 | C | C | T | SCHP | TTG -> TTA; L -> L | C | T | C | T | T | T | T | T | T | T | C |
| 1908919 | C | C | T | transaldolase | GTA -> ATA; V -> I | C | C | C | C | C | C | C | C | C | C | C |
| 1935266 | - | - | T | SCHP | frameshift in HOU | T | T | T | T | X | X | X | T | X | X | X |
| 1944910 | G | G | A | IGR upstream of *splA* |  | G | G | G | G | X | G | G | G | G | G | G |
| 1958687 | T | X | C | IGR upstream of *lukE* |  | T | T | T | T | X | T | T | T | T | T | T |
| 1960253 | A | A | T | IGR downstream hypo and tRNA | | A | A | A | A | A | A | A | A | A | A | A |
| 1962103 | T | C | C | tRNA met |  | T | C | T | C | C | C | C | C | C | C | T |
| 1966202 | C | T | T | uroporphyrinogen decarboxylase | GGA -> GAA; G -> E | T | T | T | T | T | T | T | T | T | T | T |
| 1984384 | C | T | T | RNA pseudouridylate synthase | ATC -> ATT; I -> I | T | T | T | T | T | T | T | T | T | T | T |
| 2002780 | G | G | A | 16S rRNA |  | A | A | A | A | A | A | A | A | G | G | G |
| 2027614 | A | G | G | sensor histidine kinase VraS | CTT -> CTC; L -> L | G | G | G | G | A | G | G | G | G | G | G |
| 2083248 | C | T | T | map-w protein | AAG -> AAA; K -> K | T | T | T | T | T | T | T | T | T | T | T |
| 2100795 | C | C | A | phi 77 phage tape measure protein | AGT -> ATT; S -> S | X | X | X | X | C | C | C | X | C | C | C |
| 2123815 | - | - | A | CH phage protein | frameshift in HOU | X | X | - | X | - | X | - | X | X | X | - |
| 2179818 | A | X | G | 16S rRNA |  | G | G | A | G | G | G | G | G | G | G | G |
| 2231162 | T | T | C | CHP | AAA -> AAG; K -> K | T | T | T | T | T | T | T | T | T | T | T |
| 2246738 | A | G | G | GNAT acetyltransferase | ATA -> ACA; I -> T | G | G | G | G | G | G | G | G | G | G | G |
| 2256979 | T | C | C | IGR upstream of stress protein | | C | C | C | C | C | C | C | A | C | C | C |
| 2276958 | G | G | A | PTS mannitol porter component IIA | GGA -> GAA; G -> E | G | G | G | G | G | G | G | G | G | G | G |
| 2296128 | A | X | G | 16S rRNA |  | G | A | G | A | G | G | G | G | G | G | G |
| 2322850 | C | C | T | IGR upstream of betaine transporter | | C | C | C | C | X | C | C | C | C | C | C |
| 2388859 | A | C | C | SCHP | TAT -> TCT; Y -> S | C | C | C | C | C | C | C | C | C | C | C |
| 2496166 | G | A | A | IGR upstream of SCHP | | A | A | A | A | A | A | A | A | A | A | A |
| 2591189 | A | C | C | IGR downstream MFS transporter | | C | C | C | C | C | C | C | C | C | C | C |
| 2623921 | C | A | A | cell wall surface anchor protein | CCG -> CCT; P -> P | A | X | A | X | X | A | A | X | A | A | A |
| 2627270 | - | - | TATA | IGR between *sarT* and *sarU* | shifts sar box closer to -10 | - | - | - | - | X | - | - | - | - | - | - |
| 2686085 | G | G | T | Clp protease | GCT -> TCT; A -> S | G | G | G | G | G | G | G | G | G | G | G |
| 2690233 | G | G | T | SCHP | CAA -> AAA; Q -> K | G | G | G | G | G | G | G | G | G | G | G |
| 2708710 | G | G | T | secretory antigen | GCC -> GCA; A -> A | G | G | G | G | G | G | G | G | G | G | G |
|  |  |  |  |  |  |  |  |  |  |  |  |  |  |  |  |  |
| **Sequencing errors in FPR3757 (not SNPs)** | | | | | |  |  |  |  |  |  |  |  |  |  |  |
| 34179 | A | G | T (A) | *orfX* |  | T | T | G | A | A | A | A | X | A | A | G |
| 425728 | T | A | A (T) | possible phosphogylcerate mutase |  | A | A | A | A | A | A | A | A | A | A | A |
| 517984 | C | C | T (C) | 5S rRNA |  | T | C | C | C | C | C | C | C | C | C | C |
| 517996 | A | G | G (A) | 5S rRNA |  | G | G | G | G | G | G | G | G | G | G | G |
| 555975 | C | C | T (C) | IGR upstream 5S rRNA | | T | T | T | T | T | T | T | T | T | T | T |
| 556184 | C | X | T (C) | 5S rRNA |  | T | T | T | T | T | T | T | T | T | T | T |
| 556242 | A | A | G (A) | 5S rRNA |  | G | A | A | A | A | A | A | A | A | A | A |
| 556243 | T | T | C (T) | 5S rRNA |  | C | T | T | T | T | T | T | T | T | T | T |
| 556248 | C | C | T (C) | 5S rRNA |  | T | C | C | C | C | C | C | C | C | C | C |
| 556251 | A | A | G (A) | 5S rRNA |  | G | A | A | A | A | A | A | A | A | A | A |
| 556254 | A | A | C (A) | 5S rRNA |  | C | A | A | A | A | A | A | A | A | A | A |
| 556259 | A | A | G (A) | 5S rRNA |  | G | A | A | A | A | A | A | A | A | A | A |
| 556260 | G | G | A (G) | 5S rRNA |  | A | G | G | G | G | G | G | G | G | G | G |
| 556265 | G | G | A (G) | 5S rRNA |  | A | G | G | G | G | G | G | G | G | G | G |
| 556275 | A | A | T (A) | 5S rRNA |  | T | A | A | A | A | A | A | A | A | A | A |
| 556280 | T | T | C (T) | 5S rRNA |  | C | T | T | T | T | T | T | T | T | T | T |
| 556281 | C | C | T (C) | 5S rRNA |  | T | C | C | C | C | C | C | C | C | C | C |
| 556283 | T | T | G (T) | 5S rRNA |  | G | T | T | T | T | T | T | T | T | T | T |
| 594402 | A | A | T (A) | S7 |  | A | A | A | A | A | A | A | A | A | A | A |
| 594403 | - | - | A (-) | S7 |  | - | - | - | - | - | - | - | - | - | - | - |
| 656886 | T | T | A (T) | SCHP |  | T | T | T | T | T | T | T | T | T | T | T |
| 656887 | T | T | A (T) | SCHP |  | T | T | T | T | T | T | T | T | T | T | T |
| 656890 | T | T | G (T) | SCHP |  | T | T | T | T | T | T | T | T | T | T | T |
| 659005 | T | T | A (T) | IGR upstream of hypothetical and SCHP | | T | T | T | T | X | C | C | X | T | T | T |
| 659007 | A | A | T (A) | IGR upstream of hypothetical and SCHP | | A | A | A | A | X | A | A | A | A | A | A |
| 659210 | G | G | T (G) | frameshift of SCHP |  | G | G | G | G | X | X | X | A | A | A | G |
| 659211 | A | A | T (A) | frameshift of SCHP |  | A | A | A | A | X | X | X | A | A | A | A |
| 659212 | G | G | T (G) | frameshift of SCHP |  | G | G | G | G | X | X | X | A | A | A | G |
| 659213 | C | C | T (C) | frameshift of SCHP |  | C | C | C | C | X | X | X | C | C | C | C |
| 659214 | C | C | T (C) | frameshift of SCHP |  | C | C | C | C | X | X | X | C | C | C | C |
| 659216 | G | G | T (G) | frameshift of SCHP |  | G | G | G | G | X | X | X | G | G | G | G |
| 958640 | T | T | A (T) | IGR upstream of argininosuccinate  synthase |  | T | T | T | T | T | T | T | T | T | T | T |
| 1367975 | T | X | C (T) | CHP |  | C | C | C | C | C | C | C | C | C | C | C |
| 1369691 | A | A | T (A) | CHP |  | T | A | A | A | A | A | A | A | A | A | A |
| 1740889 | A | G | G (A) | N-acetylmuramoyl-L-alanine amidase |  | G | G | G | G | G | G | G | G | G | G | G |
| 1940192 | G | G | C (G) | serine protease SplF |  | G | G | G | G | G | G | G | G | G | G | G |
| 1995662 | - | - | A (-) | transposase |  | A | X | A | X | X | A | A | X | A | A | A |
| 2179569 | T | T | C (T) | 23S rRNA |  | C | C | C | C | C | C | C | C | C | C | C |
| 2179571 | C | C | A (C) | 23S rRNA |  | A | A | A | A | A | A | A | A | A | A | A |
| 2179573 | A | A | T (A) | 23S rRNA |  | T | T | T | T | T | T | T | T | T | T | T |
| 2179574 | C | C | T (C) | 23S rRNA |  | T | T | T | T | T | T | T | T | T | T | T |
| 2179575 | A | A | T (A) | 23S rRNA |  | T | T | T | T | T | T | T | T | T | T | T |
| 2179576 | G | G | T (G) | 23S rRNA |  | T | T | T | T | T | T | T | T | T | T | T |
| 2179577 | G | G | T (G) | 23S rRNA |  | A | A | A | A | A | A | A | A | A | A | A |
| 2295821 | C | C | T (C) | 23S rRNA |  | T | C | C | C | C | C | C | C | C | C | T |
| 2295879 | T | T | C (T) | 23S rRNA |  | C | X | C | X | C | C | C | X | C | C | C |
| 2295881 | C | C | A (C) | 23S rRNA |  | A | X | A | X | A | A | A | X | A | A | A |
| 2295883 | A | A | T (A) | 23S rRNA |  | T | X | T | X | T | T | T | X | T | T | T |
| 2295884 | C | C | T (C) | 23S rRNA |  | C | X | C | X | C | C | C | X | C | C | C |
| 2295885 | A | A | T (A) | 23S rRNA |  | T | X | T | X | T | T | T | X | T | T | T |
| 2295886 | G | G | T (G) | 23S rRNA |  | T | X | T | X | T | T | T | X | T | T | T |
| 2295887 | G | G | A (G) | 23S rRNA |  | A | X | A | X | A | A | A | X | A | A | A |

Bases coloured in red match the USA300-HOU allele and those in blue match the FPR3757 allele; bases in black match neither.

*Bases in parentheses represent the base determined by resequencing.

CHP, conserved hypothetical protein; SCHP, staphylococcal conserved hypothetical protein; IGR, intergenic region. X, region missing, -, base missing
