# Supplementary material for: Subtle genetic changes enhance virulence of methicillin resistant and sensitive Staphylococcus aureus
Source: BMC Microbiol. 2007 Nov 6;7:99. doi: 10.1186/1471-2180-7-99 (PMC2222628; doi:10.1186/1471-2180-7-99)
Supplement: Additional file 3 — Predicted non-classically secreted proteins. [file 1471-2180-7-99-S3.doc]

**Supplemental Table 3 - Predicted non-classically secreted proteins.**

| **SecP score*** | **Start** | **Stop** | **Definition** |
| --- | --- | --- | --- |
| 0.982306 | 2260027 | 2260266 | staphylococcal conserved hypothetical membrane protein |
| 0.981220 | 280905 | 281078 | staphylococcal conserved hypothetical protein |
| 0.980866 | 122881 | 123762 | possible DMT superfamily drug/metabolite transporter |
| 0.980769 | 1909194 | 1909496 | staphylococcal conserved hypothetical membrane protein |
| 0.980767 | 2693406 | 2693573 | staphylococcal conserved hypothetical protein |
| 0.980720 | 2555898 | 2556284 | conserved hypothetical membrane protein |
| 0.980434 | 2854715 | 2855623 | DMT superfamily drug/metabolite transporter |
| 0.980242 | 883528 | 883788 | staphylococcal conserved hypothetical membrane protein |
| 0.980080 | 1180049 | 1180996 | TDT family tellurite/dicarboxylate transporter |
| 0.980008 | 2458606 | 2458932 | conserved hypothetical membrane protein |
| 0.980006 | 2445072 | 2445641 | staphylococcal conserved hypothetical membrane protein |
| 0.979914 | 1910114 | 1910467 | camphor resistance protein |
| 0.979900 | 2653467 | 2653619 | staphylococcal conserved hypothetical protein |
| 0.979896 | 984750 | 984935 | staphylococcal conserved hypothetical protein |
| 0.979776 | 805936 | 806802 | DMT superfamily drug/metabolite transporter |
| 0.979745 | 404232 | 405365 | probable bacterial low temperature requirement protein A |
| 0.979557 | 1532920 | 1533507 | conserved hypothetical membrane protein |
| 0.979508 | 2858775 | 2859287 | conserved hypothetical membrane protein |
| 0.979469 | 768247 | 769413 | MFS family major facilitator transporter |
| 0.979339 | 2303249 | 2303935 | probable hemolysin III |
| 0.979321 | 315754 | 316635 | DMT superfamily drug/metabolite transporter |
| 0.979083 | 2080356 | 2080532 | staphylococcal conserved hypothetical protein |
| 0.978866 | 928192 | 928344 | staphylococcal conserved hypothetical protein |
| 0.978805 | 424734 | 424985 | conserved hypothetical membrane protein |
| 0.978754 | 783267 | 783851 | staphylococcal conserved hypothetical membrane protein |
| 0.978638 | 2227458 | 2227811 | staphylococcal conserved hypothetical protein |
| 0.978594 | 1772117 | 1772932 | possible cytochrome c assembly protein |
| 0.978565 | 2380010 | 2380327 | staphylococcal conserved hypothetical membrane protein |
| 0.978559 | 1045875 | 1046813 | 1,4-dihydroxy-2-naphthoate octaprenyltransferase |
| 0.978400 | 2572074 | 2573474 | MFS family major facilitator transporter |
| 0.978328 | 436945 | 438213 | NCS2 family nucleobase:cation symporter-2 |
| 0.978241 | 2378560 | 2379486 | AEC family malonate efflux carrier |
| 0.978241 | 2508519 | 2509688 | MFS family major facilitator transporter, nitrate:nitrite antiporter |
| 0.978198 | 1746917 | 1747039 | hypothetical protein |
| 0.978144 | 1319804 | 1320295 | conserved hypothetical protein |
| 0.978078 | 2301729 | 2303072 | MFS family major facilitator transporter |
| 0.978072 | 1127825 | 1128736 | protoheme IX farnesyltransferase |
| 0.978017 | 2647542 | 2648705 | MFS family major facilitator transporter |
| 0.977953 | 2558595 | 2559806 | MFS family major facilitator transporter, bicyclomycin:cation symporter |
| 0.977943 | 766532 | 766816 | staphylococcal conserved hypothetical protein |
| 0.977907 | 346186 | 346404 | staphylococcal conserved hypothetical protein |
| 0.977905 | 157685 | 159037 | MFS family major facilitator transporter, tetracycline:cation symporter |
| 0.977851 | 426058 | 426441 | staphylococcal conserved hypothetical membrane protein |
| 0.977776 | 2432300 | 2433037 | conserved hypothetical membrane protein |
| 0.977738 | 1539287 | 1539487 | staphylococcal conserved hypothetical protein |
| 0.977696 | 749073 | 750293 | MFS family major facilitator transporter, sugar:cation symporter |
| 0.977536 | 1502421 | 1503812 | MFS family major facilitator transporter |
| 0.977529 | 1928347 | 1928523 | staphylococcal conserved hypothetical protein |
| 0.977510 | 1888582 | 1889763 | MFS family major facilitator transporter |
| 0.977477 | 393637 | 394632 | conserved hypothetical membrane protein |
| 0.977440 | 1907146 | 1907781 | conserved hypothetical protein |
| 0.977409 | 2555657 | 2555764 | staphylococcal conserved hypothetical protein |
| 0.977390 | 1019791 | 1020981 | MFS family major facilitator transporter |
| 0.977373 | 910538 | 911389 | conserved hypothetical membrane protein |
| 0.977288 | 98658 | 98861 | staphylococcal conserved hypothetical protein |
| 0.977286 | 885444 | 886061 | LysE family L-lysine exporter |
| 0.977248 | 71552 | 72973 | arginine/ornithine APC family amino acid-polyamine-organocation transporter, antiporter |
| 0.977111 | 673776 | 674762 | iron (Fe3+) ABC superfamily ATP binding cassette transporter, membrane protein |
| 0.977041 | 741519 | 742163 | conserved hypothetical membrane protein |
| 0.976948 | 1171946 | 1172092 | staphylococcal conserved hypothetical protein |
| 0.976887 | 2591330 | 2592523 | MFS family major facilitator transporter |
| 0.976872 | 837270 | 837506 | conserved hypothetical membrane protein |
| 0.976743 | 1190294 | 1191259 | phospho-N-acetylmuramoyl-pentapeptide-transferase |
| 0.976738 | 762795 | 763082 | staphylococcal conserved hypothetical membrane protein |
| 0.976712 | 1708286 | 1708555 | staphylococcal conserved hypothetical membrane protein |
| 0.976660 | 982131 | 983249 | staphylococcal conserved hypothetical membrane protein |
| 0.976644 | 2829120 | 2830172 | intercellular adhesion protein C |
| 0.976631 | 2078138 | 2078818 | staphylococcal conserved hypothetical membrane protein |
| 0.976578 | 2779296 | 2780732 | APC family amino acid-polyamine-organocation transporter |
| 0.976257 | 2387054 | 2388265 | MFS family major facilitator transporter |
| 0.976257 | 2669101 | 2670063 | DMT superfamily drug/metabolite transporter |
| 0.976241 | 1352947 | 1353135 | staphylococcal conserved hypothetical protein |
| 0.976191 | 2377278 | 2378141 | DMT superfamily drug/metabolite transporter |
| 0.976112 | 328545 | 329132 | staphylococcal conserved hypothetical membrane protein |
| 0.976101 | 99278 | 100498 | probable MFS family major facilitator transporter |
| 0.976038 | 1992832 | 1993893 | conserved hypothetical protein |
| 0.975879 | 640008 | 640376 | conserved hypothetical membrane protein |
| 0.975846 | 917571 | 917885 | conserved hypothetical membrane protein |
| 0.975842 | 151170 | 152408 | staphylococcal conserved hypothetical membrane protein |
| 0.975822 | 2535929 | 2537368 | MFS family major facilitator transporter, multidrug :cation symporter |
| 0.975737 | 1801667 | 1803028 | APC family amino acid-polyamine-organocation transporter |
| 0.975705 | 15441 | 15770 | probable membrane protein |
| 0.975562 | 319310 | 320695 | MFS family major facilitator transporter |
| 0.975547 | 2865785 | 2866180 | conserved hypothetical membrane protein |
| 0.975541 | 825209 | 826264 | conserved hypothetical membrane protein |
| 0.975522 | 183061 | 184227 | capsular polysaccharide biosynthesis protein Cap5J |
| 0.975508 | 117064 | 118452 | possible MFS family major facilitator transporter |
| 0.975224 | 2562326 | 2563735 | APC family amino acid-polyamine-organocation transporter |
| 0.975191 | 669101 | 669208 | staphylococcal conserved hypothetical protein |
| 0.975185 | 2299393 | 2300838 | MFS family major facilitator transporter |
| 0.975129 | 822352 | 822846 | conserved hypothetical protein |
| 0.975123 | 345439 | 346050 | staphylococcal conserved hypothetical membrane protein |
| 0.975087 | 2866248 | 2866601 | conserved hypothetical membrane protein |
| 0.975084 | 344609 | 345292 | staphylococcal conserved hypothetical membrane protein |
| 0.975082 | 1066669 | 1066959 | possible cytochrome c oxidase subunit IV |
| 0.975073 | 2080775 | 2080873 | staphylococcal conserved hypothetical protein |
| 0.975072 | 389330 | 390688 | MOP superfamily multidrug/oligosaccharidyl-lipid/polysaccharide flippase transporter |
| 0.975052 | 1854757 | 1854870 | hypothetical protein |
| 0.974994 | 348765 | 349448 | staphylococcal conserved hypothetical protein |
| 0.974990 | 2845365 | 2845919 | conserved hypothetical protein |
| 0.974964 | 2677257 | 2677652 | conserved hypothetical membrane protein |
| 0.974947 | 945637 | 948042 | CPA3 family monovalent cation:proton (H+) antiporter-3 subunit A |
| 0.974866 | 309042 | 309743 | murein hydrolase regulator LrgB |
| 0.974855 | 1096207 | 1097226 | cytochrome d ubiquinol oxidase subunit II |
| 0.974829 | 1468614 | 1468835 | conserved hypothetical protein |
| 0.974756 | 1044892 | 1045107 | conserved hypothetical protein |
| 0.974710 | 365095 | 366627 | SSS family solute:sodium (Na+) symporter |
| 0.974709 | 652607 | 652708 | staphylococcal conserved hypothetical protein |
| 0.974692 | 970983 | 971372 | conserved hypothetical membrane protein |
| 0.974690 | 201913 | 203163 | MFS family major facilitator transporter, multidrug cation symporter or antiporter |
| 0.974657 | 2399940 | 2400494 | biotin biosynthesis protein |
| 0.974540 | 631876 | 633276 | MFS family major facilitator transporter, proline/betaine:cation symporter |
| 0.974526 | 2438324 | 2439241 | BASS family bile acid:sodium (Na+) symporter |
| 0.974512 | 217059 | 218414 | probable LIVCS family branched chain amino acid:cation symporter |
| 0.974497 | 755637 | 756512 | undecaprenol kinase |
| 0.974405 | 2314076 | 2315269 | MFS family major facilitator transporter |
| 0.974323 | 14749 | 15444 | LIV-E family branched chain amino acid exporter |
| 0.974284 | 699178 | 700674 | CPA3 family monovalent cation (K+ |
| 0.974264 | 482432 | 482848 | staphylococcal conserved hypothetical protein |
| 0.974264 | 960601 | 961176 | conserved hypothetical membrane protein |
| 0.974229 | 1947118 | 1947879 | epidermin ABC superfamily ATP binding cassette transporter, membrane protein |
| 0.974111 | 661537 | 662172 | staphylococcal conserved hypothetical protein |
| 0.974097 | 2063109 | 2064671 | DASS family divalent anion:sodium (Na+) symporter |
| 0.974032 | 2436778 | 2437134 | staphylococcal conserved hypothetical membrane protein |
| 0.974027 | 706336 | 707172 | ABC superfamily ATP binding cassette transporter, membrane protein |
| 0.973941 | 2620847 | 2621155 | staphylococcal conserved hypothetical membrane protein |
| 0.973835 | 921445 | 922272 | conserved hypothetical membrane protein |
| 0.973831 | 308606 | 309049 | murein hydrolase regulator LrgA |
| 0.973831 | 803570 | 805075 | POT family proton (H+)-dependent oligopeptide transporter |
| 0.973820 | 728219 | 729199 | conserved hypothetical membrane protein |
| 0.973820 | 2483683 | 2484138 | conserved hypothetical membrane protein |
| 0.973770 | 701154 | 701456 | CPA3 family monovalent cation (K+ |
| 0.973738 | 2154426 | 2155676 | AMT family ammonium or ammonia transporter |
| 0.973704 | 698422 | 698847 | CPA3 family monovalent cation (K+ |
| 0.973677 | 1759767 | 1760051 | staphylococcal conserved hypothetical membrane protein |
| 0.973675 | 1166378 | 1166884 | staphylococcal conserved hypothetical membrane protein |
| 0.973669 | 642123 | 642575 | conserved hypothetical protein |
| 0.973656 | 2742634 | 2743050 | staphylococcal conserved hypothetical membrane protein |
| 0.973567 | 1555410 | 1555955 | conserved hypothetical membrane protein |
| 0.973565 | 145771 | 146178 | staphylococcal conserved hypothetical membrane protein |
| 0.973539 | 355392 | 356699 | LIVCS family branched chain amino acid:cation symporter |
| 0.973492 | 444637 | 444996 | conserved hypothetical membrane protein |
| 0.973410 | 125453 | 125926 | conserved hypothetical membrane protein |
| 0.973295 | 1177653 | 1179209 | arginine/ornithine APC family amino acid-polyamine-organocation transporter, antiporter |
| 0.973293 | 1503969 | 1505291 | APC family amino acid-polyamine-organocation transporter |
| 0.973251 | 477477 | 478961 | NADH dehydrogenase (ubiquinone) |
| 0.973200 | 494220 | 495002 | conserved hypothetical protein |
| 0.973197 | 327858 | 328511 | staphylococcal conserved hypothetical membrane protein |
| 0.973068 | 1126474 | 1127544 | cytochrome oxidase assembly protein |
| 0.973048 | 2206437 | 2207639 | FtsW/RodA/SpoVE family cell division protein |
| 0.972981 | 929814 | 931028 | D-alanine transfer protein DltB |
| 0.972976 | 91900 | 92655 | conserved hypothetical membrane protein |
| 0.972969 | 1384637 | 1384933 | conserved hypothetical protein |
| 0.972967 | 254858 | 256255 | MFS family major facilitator transporter, hexose phosphate:cation symporter |
| 0.972911 | 1946423 | 1947121 | lantibiotic epidermin immunity protein F |
| 0.972907 | 640497 | 641981 | APC family amino acid-polyamine-organocation transporter |
| 0.972895 | 2653738 | 2654817 | conserved hypothetical membrane protein |
| 0.972861 | 108248 | 108730 | staphylococcal conserved hypothetical membrane protein |
| 0.972836 | 2810172 | 2811383 | Sec family Type I general secretory pathway protein SecY |
| 0.972791 | 60794 | 61162 | conserved hypothetical membrane protein |
| 0.972750 | 2770804 | 2771004 | staphylococcal conserved hypothetical protein |
| 0.972712 | 1128761 | 1129222 | conserved hypothetical membrane protein |
| 0.972670 | 1766879 | 1767952 | possible ammonia monooxygenase |
| 0.972665 | 654587 | 655213 | staphylococcal conserved hypothetical protein |
| 0.972655 | 104170 | 104772 | staphylococcal conserved hypothetical membrane protein |
| 0.972497 | 817247 | 817375 | hypothetical protein |
| 0.972450 | 2135507 | 2136814 | Trk family potassium (K+) transporter, ABC protein |
| 0.972401 | 152398 | 153828 | MOP superfamily multidrug/oligosaccharidyl-lipid/polysaccharide flippase transporter |
| 0.972382 | 945216 | 945644 | CPA3 family monovalent cation:proton (H+) antiporter-3 subunit B |
| 0.972318 | 863811 | 864269 | staphylococcal conserved hypothetical membrane protein |
| 0.972231 | 2372441 | 2373775 | NCS2 family nucleobase:cation symporter-2 |
| 0.972185 | 1026156 | 1026959 | conserved hypothetical membrane protein |
| 0.972157 | 1443035 | 1443427 | IS1272 transposase |
| 0.972112 | 1437737 | 1439002 | diaminopimelate decarboxylase |
| 0.972104 | 1665799 | 1666671 | ABC superfamily ATP binding cassette transporter, membrane protein |
| 0.972067 | 2494206 | 2494637 | conserved hypothetical membrane protein |
| 0.972059 | 2463496 | 2464704 | ESS family glutamate:sodium (Na+) symporter |
| 0.971805 | 943386 | 944882 | CPA3 family monovalent cation:proton (H+) antiporter-3 subunit D |
| 0.971741 | 1121090 | 1122328 | probable FtsW/RodA/SpoVE family cell division protein |
| 0.971709 | 1794140 | 1795642 | conserved hypothetical membrane protein |
| 0.971651 | 1909674 | 1910117 | camphor resistance protein CrcB |
| 0.971621 | 656760 | 657389 | staphylococcal conserved hypothetical protein |
| 0.971583 | 1066956 | 1067561 | quinol oxidase subunit III |
| 0.971549 | 987482 | 987853 | staphylococcal conserved hypothetical membrane protein |
| 0.971478 | 2526427 | 2527134 | FNT family formate-nitrite transporter |
| 0.971476 | 1326251 | 1327069 | MIP family major intrinsic protein channel protein |
| 0.971420 | 1875205 | 1876866 | polysaccharide biosynthesis protein |
| 0.971410 | 474308 | 474502 | staphylococcal conserved hypothetical protein |
| 0.971394 | 370579 | 371940 | conserved hypothetical membrane protein |
| 0.971369 | 750290 | 750778 | conserved hypothetical membrane protein |
| 0.971357 | 2361013 | 2362305 | Sec family Type I general secretory pathway preprotein translocase SecY |
| 0.971257 | 2085696 | 2085875 | staphylococcal conserved hypothetical protein |
| 0.971193 | 1820698 | 1820796 | staphylococcal conserved hypothetical protein |
| 0.971181 | 2089457 | 2089609 | hypothetical protein |
| 0.971127 | 766813 | 767430 | conserved hypothetical membrane protein |
| 0.971093 | 696033 | 698435 | CPA3 family monovalent cation (K+ |
| 0.970937 | 191022 | 191405 | conserved hypothetical membrane protein |
| 0.970869 | 657585 | 658205 | staphylococcal conserved hypothetical protein |
| 0.970724 | 2404448 | 2405362 | UT family urea transporter |
| 0.970704 | 2840870 | 2840977 | hypothetical protein |
| 0.970657 | 1971544 | 1972101 | conserved hypothetical membrane protein |
| 0.970596 | 399784 | 400440 | Tat family twin arginine targeting transporter TatC |
| 0.970561 | 658336 | 658977 | staphylococcal conserved hypothetical protein |
| 0.970531 | 2014361 | 2015197 | teichoic acid ABC superfamily ATP binding cassette transporter, membrane protein |
| 0.970438 | 2746081 | 2746377 | staphylococcal conserved hypothetical protein |
| 0.970303 | 2662569 | 2663300 | conserved hypothetical protein |
| 0.970297 | 708755 | 709507 | staphylococcal conserved hypothetical membrane protein |
| 0.970211 | 880163 | 880849 | micrococcal nuclease |
| 0.970191 | 1760802 | 1761509 | A24 family peptidase |
| 0.970123 | 1647352 | 1647570 | staphylococcal conserved hypothetical membrane protein |
| 0.970062 | 2645010 | 2646287 | MFS family major facilitator transporter, glucarate:cation symporter |
| 0.970052 | 1340869 | 1340979 | staphylococcal conserved hypothetical protein |
| 0.969994 | 2713510 | 2714565 | conserved hypothetical membrane protein |
| 0.969958 | 1087107 | 1087682 | conserved hypothetical membrane protein |
| 0.969949 | 854840 | 855469 | staphylococcal conserved hypothetical protein |
| 0.969948 | 1417878 | 1418864 | oligopeptide ABC superfamily ATP binding cassette transporter, membrane protein |
| 0.969912 | 2320998 | 2322560 | BCCT family betaine/carnitine/choline transporter |
| 0.969840 | 482886 | 483560 | probable PAP2 superfamily phosphatase |
| 0.969792 | 2772686 | 2772787 | staphylococcal conserved hypothetical protein |
| 0.969763 | 1158740 | 1159354 | succinate dehydrogenase, cytochrome b558 subunit |
| 0.969720 | 2305182 | 2305697 | conserved hypothetical membrane protein |
| 0.969629 | 1781224 | 1782717 | APC family amino acid-polyamine-organocation transporter |
| 0.969565 | 2156168 | 2157247 | conserved hypothetical membrane protein |
| 0.969389 | 1825877 | 1826647 | conserved hypothetical protein |
| 0.969379 | 655339 | 655980 | staphylococcal conserved hypothetical protein |
| 0.969330 | 2024951 | 2026168 | probable ribonuclease BN |
| 0.969327 | 401656 | 402369 | staphylococcal conserved hypothetical membrane protein |
| 0.969272 | 942612 | 942905 | CPA3 family monovalent cation:proton (H+) antiporter-3 subunit F |
| 0.969266 | 1923725 | 1924375 | conserved hypothetical protein |
| 0.969158 | 812824 | 813780 | iron (Fe+3) ABC superfamily ATP binding cassette transporter, membrane protein |
| 0.969096 | 660850 | 661500 | conserved hypothetical protein |
| 0.969059 | 1757055 | 1757585 | staphylococcal conserved hypothetical protein |
| 0.969033 | 660106 | 660735 | conserved hypothetical protein |
| 0.968997 | 2458981 | 2459166 | staphylococcal conserved hypothetical protein |
| 0.968952 | 2473396 | 2474604 | MFS family major facilitator transporter, teicoplanin:cation symporter |
| 0.968930 | 2076529 | 2077269 | staphylococcal conserved hypothetical membrane protein |
| 0.968921 | 2676575 | 2677264 | conserved hypothetical membrane protein |
| 0.968891 | 698844 | 699188 | CPA3 family monovalent cation (K+ |
| 0.968807 | 2650287 | 2651057 | ABC superfamily ATP binding cassette transporter, membrane protein |
| 0.968624 | 1142346 | 1143122 | possible iron (Fe3+) ABC superfamily ATP binding cassette transporter, membrane protein |
| 0.968608 | 939110 | 940426 | NhaC family sodium:proton (Na+:H+) antiporter |
| 0.968604 | 2045254 | 2046792 | SSS family solute:sodium (Na+)/proline symporter |
| 0.968348 | 2142622 | 2143365 | conserved hypothetical membrane protein |
| 0.968296 | 2469903 | 2471834 | MFS family major facilitator transporter, multidrug:cation symporter |
| 0.968233 | 1067551 | 1069539 | quinol oxidase subunit I |
| 0.968222 | 2590357 | 2591106 | staphylococcal conserved hypothetical protein |
| 0.968204 | 1967792 | 1969015 | ABC superfamily ATP binding cassette transporter, membrane protein |
| 0.968192 | 2147579 | 2148148 | accessory gene regulator protein B |
| 0.968182 | 811824 | 812837 | iron (Fe+3) ABC superfamily ATP binding cassette transporter, membrane protein |
| 0.968126 | 184220 | 185425 | capsular polysaccharide synthesis protein Cap5K |
| 0.968037 | 82852 | 83619 | nickel (Ni2+)/peptide ABC superfamily ATP binding cassette transporter, membrane protein |
| 0.967955 | 2680480 | 2680614 | staphylococcal conserved hypothetical protein |
| 0.967806 | 2729218 | 2729493 | staphylococcal conserved hypothetical protein |
| 0.967773 | 403236 | 403865 | conserved hypothetical protein |
| 0.967627 | 723864 | 724880 | iron (Fe3+) ABC superfamily ATP binding cassette transporter, membrane protein |
| 0.967604 | 565734 | 566948 | CNT family concentrative nucleoside transporter |
| 0.967394 | 1987714 | 1987866 | staphylococcal conserved hypothetical protein |
| 0.967390 | 1113652 | 1114071 | staphylococcal conserved hypothetical membrane protein |
| 0.967385 | 387348 | 388703 | MATE family multi-antimicrobial extrusion protein |
| 0.967333 | 2729530 | 2729643 | hypothetical protein |
| 0.967309 | 2486063 | 2487661 | LctP family L-lactate permease |
| 0.966859 | 2308837 | 2309868 | probable iron (Fe3+) ABC superfamily ATP binding cassette transporter, membrane protein |
| 0.966828 | 1279125 | 1279907 | phosphatidate cytidylyltransferase |
| 0.966767 | 2454483 | 2455742 | staphylococcal conserved hypothetical membrane protein |
| 0.966701 | 2211820 | 2212575 | conserved hypothetical membrane protein |
| 0.966669 | 2636416 | 2637774 | GntP family gluconate:proton (H+) symporter |
| 0.966618 | 131129 | 132127 | iron (Fe3+) ABC superfamily ATP binding cassette transporter, membrane protein |
| 0.966570 | 1088552 | 1089847 | staphylococcal conserved hypothetical membrane protein |
| 0.966472 | 656068 | 656685 | staphylococcal conserved hypothetical protein |
| 0.966352 | 2739510 | 2740958 | APC family amino acid-polyamine-organocation transporter |
| 0.966291 | 2226454 | 2226666 | F-ATPase superfamily proton( H+)- |
| 0.966235 | 2687515 | 2687694 | conserved hypothetical protein |
| 0.966101 | 1015080 | 1016165 | conserved hypothetical membrane protein |
| 0.966066 | 2850920 | 2851687 | conserved hypothetical protein |
| 0.965847 | 2603134 | 2604672 | AbgT family p-aminobenzoyl-glutamate transporter |
| 0.965819 | 1217459 | 1217671 | staphylococcal conserved hypothetical protein |
| 0.965770 | 2419410 | 2419832 | conserved hypothetical membrane protein |
| 0.965763 | 360369 | 361400 | PfoR family transcriptional regulator |
| 0.965724 | 161929 | 162654 | ABC superfamily ATP binding cassette transporter, membrane protein |
| 0.965562 | 944875 | 945216 | CPA3 family monovalent cation:proton (H+) antiporter-3 subunit C |
| 0.965430 | 1457130 | 1457615 | IS200 transposase |
| 0.965272 | 17392 | 18330 | probable membrane protein |
| 0.965270 | 2336155 | 2336280 | staphylococcal conserved hypothetical protein |
| 0.965213 | 2582387 | 2583565 | MFS family major facilitator transporter, chloramphenicol:cation symporter |
| 0.965141 | 2852884 | 2854302 | DASS family divalent anion:sodium (Na+) symporter |
| 0.964943 | 1205688 | 1206179 | A8 family signal peptidase II |
| 0.964855 | 197947 | 198708 | ABC superfamily ATP binding cassette transporter, membrane protein |
| 0.964717 | 2435119 | 2436498 | APC family amino acid-polyamine-organocation transporter |
| 0.964670 | 2765626 | 2765739 | staphylococcal conserved hypothetical protein |
| 0.964656 | 1208231 | 1209547 | NCS family uracil:cation symporter |
| 0.964618 | 1381078 | 1383783 | aconitate hydratase |
| 0.964608 | 2606781 | 2607203 | conserved hypothetical protein |
| 0.964497 | 763365 | 764918 | DASS family divalent anion:sodium (Na+) symporter |
| 0.964228 | 1901841 | 1903133 | ArsB arsenite-antimonite efflux family transporter, membrane subunit |
| 0.964222 | 1551013 | 1552473 | elastin-binding protein |
| 0.964205 | 2514155 | 2514832 | nitrate reductase gamma subunit |
| 0.964122 | 2605658 | 2606077 | conserved hypothetical protein |
| 0.963947 | 719161 | 720390 | CNT family concentrative nucleoside transporter |
| 0.963808 | 2301157 | 2301630 | staphylococcal conserved hypothetical membrane protein |
| 0.963665 | 2754857 | 2756479 | BCCT family betaine/carnitine/choline transporter |
| 0.963505 | 1187386 | 1187787 | cell division protein |
| 0.963492 | 541678 | 542712 | MOP superfamily PST family polysaccharide transporter |
| 0.963148 | 942905 | 943384 | CPA3 family monovalent cation:proton (H+) antiporter-3 subunit E |
| 0.962997 | 1179517 | 1179744 | staphylococcal conserved hypothetical protein |
| 0.962681 | 284095 | 285624 | PTS family glucose/glucoside (glc) porter component IIABC |
| 0.962560 | 1654626 | 1654925 | staphylococcal conserved hypothetical protein |
| 0.962554 | 2252716 | 2253066 | conserved hypothetical membrane protein |
| 0.962529 | 2583970 | 2584746 | ABC superfamily ATP binding cassette transporter, membrane protein |
| 0.962303 | 277176 | 278405 | conserved hypothetical protein |
| 0.962159 | 2220116 | 2220349 | conserved hypothetical protein |
| 0.962058 | 1029519 | 1030877 | Trk family potassium (K+) transporter, membrane protein |
| 0.962039 | 2396372 | 2397043 | molybdenum (Mo2+) ABC superfamily ATP binding cassette transporter, membrane protein |
| 0.961842 | 2759628 | 2760998 | CitMHS family citrate-magnesium (Mg2+):proton (H+) citrate-calcium (Ca2+):proton (H+) symporter |
| 0.961760 | 382560 | 383906 | PTS family porter component IIC |
| 0.961707 | 2545751 | 2546218 | staphylococcal conserved hypothetical membrane protein |
| 0.961642 | 1557481 | 1557687 | staphylococcal conserved hypothetical protein |
| 0.961637 | 2320035 | 2320274 | staphylococcal conserved hypothetical membrane protein |
| 0.961600 | 1930534 | 1931007 | conserved hypothetical protein |
| 0.961321 | 2667148 | 2668191 | transcriptional regulator |
| 0.961240 | 2557754 | 2558413 | staphylococcal conserved hypothetical protein |
| 0.961058 | 126188 | 127780 | LctP family L-lactate permease |
| 0.960888 | 711688 | 712521 | teichoic acid ABC superfamily ATP binding cassette transporter, membrane protein |
| 0.960681 | 400502 | 400717 | Tat family twin arginine targeting transporter TatA |
| 0.960536 | 750877 | 751566 | staphylococcal conserved hypothetical membrane protein |
| 0.960366 | 942278 | 942634 | CPA3 family monovalent cation:proton (H+) antiporter-3 subunit G |
| 0.960265 | 2307872 | 2308840 | iron (Fe3+) ABC superfamily ATP binding cassette transporter, membrane protein |
| 0.959797 | 677097 | 677840 | staphylococcal conserved hypothetical protein |
| 0.959779 | 193965 | 194924 | CDF family cation diffusion facilitator |
| 0.959717 | 2226709 | 2227437 | F-ATPase superfamily proton( H+)- |
| 0.959642 | 2195970 | 2197646 | P-ATPase superfamily P-type ATPase potassium (K+) transporter subunit A |
| 0.959536 | 139359 | 141095 | IucA/IucC family siderophore biosynthesis protein |
| 0.959459 | 700675 | 701157 | CPA3 family monovalent cation (K+ |
| 0.959363 | 1701579 | 1702265 | probable competence protein ComEA |
| 0.959253 | 333822 | 334280 | virulence protein EssA |
| 0.959246 | 1986542 | 1986937 | staphylococcal conserved hypothetical protein |
| 0.959057 | 1928534 | 1928917 | staphylococcal conserved hypothetical protein |
| 0.959007 | 2575421 | 2576056 | glycine betaine/choline ABC superfamily ATP binding cassette transporter, membrane protein |
| 0.958664 | 2306266 | 2307636 | conserved hypothetical membrane protein |
| 0.958566 | 997736 | 998698 | oligopeptide ABC superfamily ATP binding cassette transporter, membrane protein |
| 0.958411 | 2646834 | 2647445 | alkaline phosphatase |
| 0.958051 | 2621477 | 2622670 | MFS family major facilitator transporter, cation symporter |
| 0.957869 | 701431 | 701868 | CPA3 family monovalent cation (K+ |
| 0.957802 | 249710 | 250549 | maltose/maltodextrin ABC superfamily ATP binding cassette transporter, membrane protein |
| 0.957735 | 2138275 | 2138652 | staphylococcal conserved hypothetical membrane protein |
| 0.957241 | 2443534 | 2444913 | NhaC family sodium:proton (Na+:H) antiporter |
| 0.957095 | 2440105 | 2441709 | PTS family arbutin-salicin-cellobiose (ASC) porter component IIBC |
| 0.957069 | 690106 | 690489 | staphylococcal conserved hypothetical membrane protein |
| 0.957009 | 790975 | 792915 | possible phosphatidylglycerol--membrane-oligosaccharide glycerophosphotransferase |
| 0.957008 | 1227030 | 1227308 | conserved hypothetical protein |
| 0.956819 | 2287746 | 2288678 | conserved hypothetical protein |
| 0.956772 | 354331 | 355176 | FNT family formate-nitrite transporter |
| 0.956699 | 1256028 | 1258634 | conserved hypothetical membrane protein |
| 0.956695 | 1151160 | 1151681 | probable colicin V production protein |
| 0.956602 | 248440 | 249708 | maltose/maltodextrin ABC superfamily ATP binding cassette transporter, membrane protein |
| 0.956457 | 974704 | 976518 | acyltransferase |
| 0.956453 | 254149 | 254721 | conserved hypothetical protein |
| 0.956442 | 1680493 | 1680837 | diacylglycerol kinase |
| 0.956370 | 2265637 | 2265741 | hypothetical protein |
| 0.956320 | 611689 | 614532 | Ser-Asp rich fibrinogen/bone sialoprotein-binding protein SdrC |
| 0.956274 | 2842770 | 2843603 | cobalt (Co2+) ABC superfamily ATP binding cassette transporter, membrane protein |
| 0.956222 | 2112362 | 2112598 | staphylococcal conserved hypothetical bacteriophage protein |
| 0.956199 | 238000 | 239163 | oligopeptide ABC superfamily ATP binding cassette transporter, membrane protein |
| 0.956198 | 2709115 | 2710926 | probable acetyltransferase |
| 0.956152 | 1422352 | 1423203 | phosphate ABC superfamily ATP binding cassette transporter, ABC protein |
| 0.956075 | 225822 | 227276 | probable PTS family sucrose porter component IIBC |
| 0.955879 | 1441631 | 1442767 | tellurite resistance protein |
| 0.955685 | 998710 | 999591 | oligopeptide ABC superfamily ATP binding cassette transporter, membrane protein |
| 0.955594 | 2722854 | 2724194 | FeoB family ferrous iron (Fe2+) uptake protein |
| 0.955587 | 494999 | 496117 | conserved hypothetical membrane protein |
| 0.955514 | 1733051 | 1733197 | staphylococcal conserved hypothetical protein |
| 0.955468 | 2471847 | 2472494 | conserved hypothetical protein |
| 0.955349 | 2126085 | 2126267 | staphylococcal conserved hypothetical bacteriophage protein |
| 0.955328 | 236558 | 237994 | oligopeptide ABC superfamily ATP binding cassette transporter, membrane protein |
| 0.955220 | 2469019 | 2469657 | staphylococcal conserved hypothetical membrane protein |
| 0.955181 | 1168797 | 1168982 | staphylococcal conserved hypothetical protein |
| 0.955151 | 1844583 | 1844675 | hypothetical protein |
| 0.954964 | 2416585 | 2417985 | NhaC family sodium:proton (Na+:H) antiporter |
| 0.954954 | 2566030 | 2566131 | staphylococcal conserved hypothetical protein |
| 0.954783 | 2856588 | 2857604 | NiCoT family nickel (Ni2+)-cobalt (Co2+) transporter |
| 0.954772 | 1084893 | 1085699 | ABC superfamily ATP binding cassette transporter, membrane protein |
| 0.954697 | 2461505 | 2462176 | conserved hypothetical membrane protein |
| 0.954503 | 662407 | 663033 | staphylococcal conserved hypothetical protein |
| 0.954133 | 844214 | 845053 | prolipoprotein diacylglycerol transferase |
| 0.954078 | 1192626 | 1193945 | cell division protein FtsQ |
| 0.953745 | 268309 | 268416 | hypothetical protein |
| 0.953643 | 2262800 | 2263780 | CDF family cation diffusion facilitator CzrB |
| 0.953632 | 1423250 | 1424167 | phosphate ABC superfamily ATP binding cassette transporter, membrane protein |
| 0.953569 | 1140382 | 1141458 | staphylococcal conserved hypothetical protein |
| 0.953338 | 788509 | 788715 | staphylococcal conserved hypothetical protein |
| 0.952691 | 492763 | 493032 | staphylococcal conserved hypothetical protein |
| 0.952633 | 2325386 | 2326255 | conserved hypothetical protein |
| 0.952401 | 1198983 | 1199273 | conserved hypothetical protein |
| 0.952170 | 2457451 | 2457564 | staphylococcal conserved hypothetical protein |
| 0.952083 | 1016507 | 1018075 | AGCS family alanine or glycine:sodium (Na+) or proton (H+) symporter |
| 0.951972 | 2477145 | 2478113 | conserved hypothetical membrane protein |
| 0.951924 | 1758627 | 1758746 | staphylococcal conserved hypothetical protein |
| 0.951888 | 2827959 | 2828264 | intercellular adhesion protein D |
| 0.951272 | 2864045 | 2864236 | staphylococcal conserved hypothetical protein |
| 0.950921 | 1514238 | 1514570 | staphylococcal conserved hypothetical protein |
| 0.950861 | 1469053 | 1469757 | conserved hypothetical protein |
| 0.950547 | 1027193 | 1029502 | S1C family peptidase |
| 0.950167 | 989022 | 990092 | oligopeptide ABC superfamily ATP binding cassette transporter, membrane protein |
| 0.950095 | 738766 | 739773 | probable PiT family inorganic phosphate transporter |
| 0.950052 | 2090978 | 2091112 | staphylococcal conserved hypothetical membrane protein |
| 0.949802 | 50912 | 51202 | staphylococcal conserved hypothetical protein |
| 0.949723 | 2609731 | 2610525 | lipoprotein |

* The SecP score was determined using the SecretomeP 2.0 Server ([www.cbs.dtu.dk/services/SecretomeP/](http://www.cbs.dtu.dk/services/SecretomeP/) [54]). The range of values is 0-1.000000. Sequences with predicted signal cleavage sequences were removed from the list.
